# Natural Products in the Management of Gastroesophageal Reflux Disease: Mechanisms, Efficacy, and Future Directions

**DOI:** 10.3390/nu17061069

**Published:** 2025-03-19

**Authors:** Kayode Komolafe, Titilope Ruth Komolafe, Olamide Olajusi Crown, Basiru Ajiboye, Felicite Noubissi, Ifedayo Victor Ogungbe, Barbara Graham

**Affiliations:** 1 Environmental Science PhD Program, Jackson State University, Jackson, MS 39217, USA; 2 Department of Biology, Jackson State University, Jackson, MS 39217, USA; 3 Chemistry and Biotechnology Science and Engineering Programs, The University of Alabama in Huntsville, Huntsville, AL 35899, USA; 4 Phytomedicine and Molecular Toxicology Research Laboratory, Department of Biochemistry, Federal University Oye Ekiti, Oye Ekiti 370112, Nigeria

**Keywords:** gastroesophageal, reflux disease, GERD, natural products, anti-inflammatory

## Abstract

Gastroesophageal reflux disease (GERD) is a common gastrointestinal disorder that is defined by the reflux of gastric contents into the esophagus, and it results in symptoms such as esophageal inflammation, regurgitation, and indigestion. Although proton pump inhibitors (PPIs) and histamine-2 receptor antagonists are frequently employed to treat GERD, their prolonged administration is associated with adverse effects, necessitating the development of alternative therapeutic strategies. Natural products are now recognized as promising candidates for the management of GERD due to their bioactive compounds, which possess antioxidant, anti-inflammatory, and mucosal-protective properties. The potential of natural products in the treatment of GERD is comprehensively examined in this review, with a focus on their mechanisms of action, which include acid suppression, esophageal mucosal regeneration, anti-inflammatory activity, and gut microbiota modulation. Also, the efficacy and safety of key natural products, including flavonoids, polyphenols, plant-derived oils, herbal extracts, probiotics, and dietary components, in preclinical and clinical studies, are assessed. Additionally, this review addresses the barriers confronting the translation of natural therapies into clinical practice, such as regulatory obstacles, variability in bioavailability, and the need for dosage standardization. The integration of natural products into the management of GERD has the potential to enhance conventional therapies, providing a more comprehensive and secure approach for patients.

## 1. Introduction

Gastroesophageal reflux disease (GERD) is a pathological condition that affects the gastrointestinal tract and is both chronic and multifactorial. One major hallmark of this disorder is the retrograde movement of gastric contents into the esophagus. Notable symptoms of GERD are heartburn, acid regurgitation, and, in severe cases, mucosal injury [1]. GERD was defined by the Montreal Consensus as a condition in which reflux causes “troublesome symptoms and/or complications” [2]. GERD is associated with significant morbidity and substantial economic toll, and annual expenses in the United States alone were once reported to exceed $10 billion [3]. Increasing risk factors such as obesity, aging, and certain lifestyle behaviors also continue to increase GERD’s prevalence. In terms of epidemiology, GERD affects people irrespective of age, but the prevalence is influenced by geography and demographics. An estimated 20–30% of the population has GERD symptoms in Western countries for instance, while the prevalence is recorded at 7.8–8.8% in East Asia [1,4,5]. Gender disparities in GERD phenotypes have been identified, with women being more likely to present with non-erosive reflux disease (NERD) and males with erosive esophagitis and Barrett’s esophagus (BE) [6]. Additional risk factors include advanced age, a body mass index (BMI) greater than 30, smoking, alcohol use, and the use of medications that reduce lower esophageal sphincter (LES) pressure [7]. GERD complications include esophagitis, peptic stricture, esophageal ulcers, and BE, which is a precancerous disease that raises the chance of esophageal adenocarcinoma. Aside from esophageal manifestations, GERD can cause extra-esophageal symptoms such as chronic cough, laryngitis, asthma, and dental erosion, thus indicating its systemic significance [8,9].

The pathogenesis of GERD is complex and both anatomical and functional impairments have been implicated. The malfunction of the esophagogastric junction (EGJ), which is responsible for preventing the retrograde movement of stomach contents, is an important feature of GERD. The weakening of the barrier occurs due to structural abnormalities such as hiatal hernia, hypotensive LES, and transitory LES relaxations [7]. The disease is then worsened by defective mucosal defenses, decreased salivary bicarbonate, and impaired esophageal clearance mechanisms which consequently prolong acid exposure and esophageal inflammation [7,10]. Emerging research has also revealed the role of inflammatory mediators and cytokines in the progression of GERD, particularly in complications such as reflux esophagitis and BE. The recruitment of inflammatory cells and tissue damage have been associated with certain pro-inflammatory pathways involving players such as hypoxia-inducible factors and nuclear factor kappa-light-chain-enhancer of activated B cells (NF-κB) [7,11]. GERD has been traditionally managed by lifestyle modifications and pharmacologic interventions, primarily by using proton pump inhibitors (PPIs). These classes of drugs are used to manage gastric acid secretion and offer mucosal healing and symptom relief in many GERD patients [4]. However, over 30% of GERD patients on PPI treatment do not react well to these medications, which results in persistent symptoms and an increased risk of side effects such as BE [12]. Long-term usage of PPIs comes with side effects such as bone fractures, renal failure, increased risk of infections, and stomach cancers [13]. Surgical procedures, such as laparoscopic anti-reflux surgery (LARS), are also used by individuals who are refractory to PPIs or who desire alternatives to pharmacological treatment. LARS has its limitations even though it is reasonably successful. About 17.7% of patients who undergo the operation require further medical or surgical intervention; about 4% report postoperative problems [12]. Additionally, the newer endoscopic procedures that are less invasive often lack robust long-term efficacy data. With these limitations in mind, there is an increasing focus on complementary and alternative therapies (CAT), such as dietary modifications, physical activity, and natural products. Lifestyle changes (including weight loss, raising the level of the bed head, and dietary changes) have shown some levels of effectiveness. Dietary modifications might be effective to a significant extent for managing symptoms of GERD. Symptoms reduction has been linked to the Mediterranean diet, which is abundant in fruits, vegetables, whole grains, and healthy lipids. The emphasis of the diet on anti-inflammatory nutrients may contribute to this advantageous outcome [14,15]. In contrast, diets high in fermentable oligosaccharides, disaccharides, monosaccharides, and polyols (FODMAPs) can worsen digestive issues [16]. The major challenges with dietary and lifestyle modifications, however, are poor compliance and non-specific guidelines.

It is necessary to conduct a thorough assessment of natural products and the extent to which they can be useful in GERD due to the persistent challenges in managing the disorder. There is an ongoing need to wholly evaluate alternative treatments. There are possibilities that some herbal extracts, polyphenols, probiotics, and some other natural substances may be able to change or impact the underlying processes of gastroesophageal reflux disease (GERD) [15,17]. These pathological processes include heightened inflammation and gastric acid production, as well as reduced mucosal defenses. Despite their potential, the effectiveness and mechanisms of these natural products remain to be fully explored, especially when considered in the light of orthodox medicines. Hence, this review will attempt to thoroughly examine the natural products that are used to treat GERD, their known mechanisms of action, clinical effectiveness, and the possibility of integration into existing therapy paradigms. The goal of this review is to explore the current literature, such that significant information gaps will be addressed and new directions for study and therapy will be suggested. This could allow for better GERD management and the development of novel, more comprehensive, and safer therapeutic options.

## 2. Clinical Complexity of GERD: Erosive and Non-Erosive Disease and Barrett’s Esophagus

GERD as a pathological condition presents diverse clinical manifestations that include erosive reflux disease, non-erosive reflux disease (NERD), and Barrett’s esophagus. Each of these phenotypes reflects varying degrees of esophageal damage and symptomatology, thereby making the diagnosis and management of GERD complex [18]. Erosive reflux disease is characterized by visible mucosal breaks on endoscopy, and it has long been the traditional focus of clinical trials evaluating proton pump inhibitors (PPIs) and H2 receptor antagonists. It is now known that it only represents a minority of GERD cases, occurring in about 30% of GERD patients. On the other hand, around 70% of GERD patients have NERD, which is characterized by classic reflux symptoms but no apparent esophageal lesions on endoscopy. The significant incidence of NERD has prompted curiosity in understanding its various subgroups and their implications for treatment [1,7]. Barrett’s esophagus (BE) is a more serious GERD complication that causes metaplastic alterations in the esophageal lining because of prolonged acid exposure. This complication increases the risk of esophageal cancer; however, it is positive that only a small fraction of NERD patients will develop BE [18]. It is crucial to identify those who are at higher risk for BE in order to allow for timely intervention. The heterogeneity of NERD is evident in its subdivisions: true NERD, reflux hypersensitivity, and functional heartburn [18]. It is important that the NERD subgroups are accurately classified to facilitate tailored management and individualized therapy. True NERD is diagnosed when patients exhibit abnormal esophageal acid exposure with a positive symptom association to acid or non-acid reflux. This form of NERD remains within the GERD spectrum and usually responds well to acid-suppressive therapy [19]. Reflux hypersensitivity encompasses individuals who have normal esophageal acid exposure but unusually high sensitivity to acid or non-acid reflux. These individuals present with typical GERD symptoms and a positive symptom association despite the lack of excessive acid exposure. The diagnosis and management of this subgroup of NERD is complicated because the disease is more of a sensory disorder rather than a true reflux disease [20]. Functional heartburn is a form of NERD that has now been excluded from the GERD realm. It involves heartburn symptoms without any evidence of acid or non-acid reflux and no response to PPI therapy. The pathological condition is now recognized as a functional esophageal disorder rather than a reflux-related issue, necessitating different therapeutic approaches like pain modulators to be used in its management [18,21]. The distinction between these NERD subgroups has been refined through 24-h impedance–pH monitoring, which evaluates both acid exposure and symptom association. This diagnostic advancement clarifies the varying pathophysiological mechanisms underlying reflux symptoms, enabling more targeted and effective treatments [18].

## 3. GERD Pathophysiology: The Role of Gastric Acid Secretion, Mucosal Injury, and Inflammatory Pathways

Gastroesophageal reflux disease (GERD) is a pathological condition that results from a combination of multiple factors. Excessive gastric acid, weakened esophageal defenses, and ongoing inflammation are some of the prominent factors already identified [1,4]. The main component of stomach acid, hydrochloric acid (HCl), also plays a vital role in damaging the esophageal lining in GERD. HCl-induced damage causes an inflammatory response that aggravates tissue injury and contributes to the chronic nature of GERD [22]. Furthermore, bile acids and pepsin exacerbate esophageal damage while chronic inflammation prolongs it. Recent research has indicated that imbalances in gut microbiota (dysbiosis) are another contributor to GERD. The condition supports the progression of GERD and associated consequences such as Barrett’s esophagus and esophageal cancer [22,23]. A detailed assessment of factors involved in the pathogenesis of GERD, including those aimed at restoring microbial balance, could help to identify possible therapeutic approaches that can reduce inflammation and protect the esophagus.

### 3.1. Gastric Acid Secretion and Its Role in Mucosal Injury

Gastric acid secretion is necessary for digestion, but the abnormal reflux of the acidic gastric contents into the esophagus, as occurs in GERD, results in mucosal injury. The acid, mainly HCl, changes the potential difference of the esophageal mucosa and generates cellular stress, which jeopardizes the cellular membrane integrity. The enhanced membrane permeability allows harmful substances such as bile acids and pepsin to penetrate the mucosa, worsening the damage [22]. Bile acids cause harm by solubilizing cell membranes and increasing hydrogen ion absorption. This causes increased acidity of the esophagus lumen and worsens mucosal damage [22,24]. Furthermore, a proteolytic enzyme, pepsin, damages esophageal epithelial cells by destroying extracellular proteins. It induces oxidative stress upon intracellular uptake, and this exacerbates tissue damage. These combined biochemical assaults produce a hostile environment that promotes inflammation and predisposes the esophagus to chronic injury [22,24].

### 3.2. Biochemical Events in Inflammatory Pathways in GERD

The inflammatory response in GERD is driven by a cascade of biochemical events that include immune cell recruitment, cytokine release, and oxidative damage. It has long been established that some pro-inflammatory mediators such as interleukin (IL)-8, platelet-activating factor (PAF), and interferon-gamma are crucially involved in the pathogenesis of GERD-related mucosal damage. GERD-affected mucosa has been reported to express a large amount of the strong neutrophil chemoattractant, interleukin-8 (IL-8) [25]. These mediators provoke tissue damage by creating reactive oxygen species (ROS) and sustaining an inflammatory microenvironment [22,26]. The recruited neutrophils produce more ROS, worsen oxidative stress, and consequently prolong mucosal damage [27]. Other interleukins such as IL-1β and IL-6 are involved in further regulation of inflammatory responses, and IL-1β levels often increase in response to acid exposure. Platelet-activating factor (PAF), a phospholipid mediator, stimulates the adhesion of eosinophil to endothelial cells and this initiates further inflammatory cascades [22]. These events contribute to chronic inflammation, tissue remodeling, and fibrosis. Furthermore, esophageal epithelial cells produce cytokines and exhibit adhesion molecules, attracting immune cells and promoting inflammation. Keratinocytes exposed to acid and bile salts promote T-cell and neutrophil chemotaxis, which exacerbates inflammation [27,28]. Mesenchymal cells also contribute to inflammatory reactions by producing pro-inflammatory cytokines and interacting with immune cells. Endothelial cells regulate leukocyte recruitment by increasing the expression of adhesion molecules such as MAdCAM-1, ICAM-1, VCAM-1, and E-selectin. This persistent inflammation in GERD has a strong relationship to esophageal remodeling and potential malignant transformation [27]. Similarly, chronic oxidative stress leads to DNA damage, genetic instability, and aberrant DNA methylation, promoting carcinogenesis [29]. Elevated ROS levels are observed in GERD, Barrett’s esophagus (BE), and esophageal adenocarcinoma. This shows that inflammation-driven oxidative stress plays a vital role in the progression of the disease [30].

### 3.3. The Contribution of Gut Microbiota to the Development of GERD

GERD and its complications, such as Barrett’s esophagus (BE) and esophageal adenocarcinoma (EAC), are significantly influenced by the gut microbiota (GM), as indicated by numerous research findings [31]. The gut microbiome influences GERD susceptibility because they are involved in the regulation of immune responses, inflammation, and esophageal barrier functions. Genome-wide association studies (GWAS) have identified specific microbial taxa linked to GERD risk [32]. GERD-induced inflammation alters esophageal microbiota, favoring gram-negative bacteria such as Proteobacteria, Fusobacteria, Spirochaetes, Rothia, and Campylobacter [33]. This dysbiosis may be the consequence of the acidic environment caused by GERD, and it further promotes disease progression [33]. A notable shift in the Bacteroidetes–Firmicutes ratio has been observed in BE patients, resembling microbiota changes seen in individuals consuming high-fat diets (HFD). HFD can modify the composition of the microbial population in the gut, cause inflammation, and promote BE and EAC development [33,34]. Mendelian randomization investigations observed that 11 bacterial taxa and 13 metabolic pathways were associated with GERD compared to 18 taxa and 5 pathways associated with BE [35]. It was found that *Faecalibacterium prausnitzii* had a substantial correlation with both conditions, while weight and body mass index (BMI) are crucial mediators [35]. Furthermore, microbiome-mediated inflammation may accelerate BE progression via lipopolysaccharide (LPS)-mediated activation of Toll-like receptor 4 (TLR-4), which leads to IL-18 production and pro-inflammatory cascades [36]. The microbiota of the precancerous consequence of GERD, Barrett’s esophagus (BE), changes considerably as the disease progresses, with the microbial makeup typically shifting from type I to type II. This change has been characterized by a rise in the prevalence of microbial populations associated with esophageal carcinogenesis, such as *Treponema denticola*, *Streptococcus mitis*, and *Streptococcus anginosus* [31,36,37]. Against this backdrop, efforts should be made in support of research aimed at harnessing GM modification for the treatment of GERD and associated consequences.

## 4. General Overview of Natural Products and Their Role in GERD Management

### 4.1. Polyphenols and Flavonoids: Antioxidant, Anti-Inflammatory and Mucosal Healing Effects

Polyphenols, which are naturally occurring chemicals of plant origin, are recognized for their antioxidant properties and prospective health benefits. Flavonoids are a subclass of polyphenols that are very much present in fruits, vegetables, teas, and herbs. It has been established that flavonoids and polyphenols could contribute significantly to GERD management by alleviating inflammation, neutralizing oxidative stress, and promoting mucosal defense [38,39]. If incorporated into dietary and therapeutic regimens, these natural antioxidants could serve as a supplement to present treatments since they appear to have the potential to improve patient outcomes and reduce adverse effects. Flavonoids’ powerful anti-inflammatory and antioxidant properties make them useful in the treatment of GERD. Studies have shown that oxidative stress contributes to mucosal damage and inflammation and aggravates clinical features of GERD [27]. Antioxidant phytochemicals like flavonoids and polyphenols could relieve the symptoms of GERD by lowering oxidative stress in the esophageal mucosa [40,41,42]. Onions and apples, which contain the flavonoid quercetin, are capable of reducing esophageal inflammation and inhibiting pro-inflammatory cytokines such as tumor necrosis factor-alpha (TNF-α) [40,42]. In the same way, polyphenols, which are abundant in green tea, berries, and red wine, function as ROS scavengers, thereby preventing the oxidation of the esophageal lining [43]. Additionally, green tea polyphenols, such as epigallocatechin gallate (EGCG), have anti-inflammatory properties and can reduce NF-κB activation, a critical regulator of inflammatory pathways associated with GERD [44]. According to [45,46], proper polyphenol intake may help maintain gut health by modulating microbiota composition since polyphenols and gut microbiota interact beneficially. Polyphenols are metabolized by gut microbiota, enhancing bioavailability while also acting as metabolic prebiotics. They could thus support GI health by inhibiting harmful microbiota and enriching beneficial ones [46,47].

Furthermore, certain polyphenols and flavonoids can stimulate mucus production and strengthen the esophageal barrier, hence improving mucosal protection. Curcumin, the active compound in turmeric, was found to promote the healing of damaged mucosa while also reducing acid secretion [42,48]. Antioxidant phytochemicals could increase the levels of endogenous antioxidants, lower pepsin and gastric acid generation, and reduce lipid peroxidation and ulceration, thereby minimizing later damage to the gastric mucosa in GERD [41].

### 4.2. Plant Oils and Extracts: Gastroprotective, Mucosal Healing, and Antioxidant Properties

Plant extracts and oils are becoming recognized for their gastroprotective and mucosal restorative effects. Most of them function through gastroprotective and mucosal-healing mechanisms while some others possess additional antioxidant and/or anti-inflammatory effects. Essential oil from *Syzygium aromaticum* (clove) and its main component, eugenol, was found to demonstrate significant gastroprotective effects linked to increased gastric mucus production rather than alterations in gastric juice volume, acidity, nitric oxide, or endogenous sulfhydryl activity [49]. *Citrus aurantium* essential oil and its primary component, limonene, significantly prevented gastrointestinal mucosal injury in rats by increasing mucus production and maintaining basal prostaglandin E2 (PGE2) levels. They, however, showed no effect on acid secretion, serum gastrin, or glutathione levels. Their ability to protect the gastric lining while not lowering acid production offers them interesting alternatives to proton pump inhibitors (PPIs) since adverse effects such as rebound acid hypersecretion in GERD are avoided [50]. Similarly, essential oils from Citrus lemon also offered significant gastroprotection linked to increased mucus secretion, vasoactive intestinal peptide (VIP), and prostaglandin E2 (PGE2) levels rather than modulation of mucosal redox factors [51]. The gastroprotective effects of *Hyptis martiusii* essential oil, however, are due to its ability to increase mucus production, enhance gastric mucosal regeneration, and produce antioxidant effects that include reducing membrane lipid peroxidation and preserving thiol groups [52]. Additionally, chamomile (*Matricaria chamomilla*) extract’s therapeutic effect is due to its anti-inflammatory and soothing properties which may help alleviate esophageal irritation and promote mucosal healing [53]. Additionally, numerous plant extracts have anti-inflammatory, antioxidant, and mucosal-protective properties. For example, Aloe vera’s anti-inflammatory and wound-healing qualities have been shown to be useful in lowering GERD symptoms. A clinical investigation revealed the ability of Aloe vera syrup to heal the mucosal as it considerably reduced heartburn, regurgitation, and nausea [54]. Similarly, licorice root (*Glycyrrhiza glabra*) extract has been demonstrated to increase mucus secretion and create a protective barrier against acid damage [55]. Deglycyrrhizinated licorice (DGL) is often used to counteract glycyrrhizin’s hypertensive effects while maintaining its gastroprotective properties Murray [56] 2020. Also, ginger extract (*Zingiber officinale*) produces prokinetic effects that improve stomach emptying and reduce acid reflux. It has been indicated that ginger’s anti-inflammatory and antioxidant components, especially gingerols and shogaols, are involved in protecting the mucosa [57,58]. These plant-based cures are indicators of the potential of natural products in GERD therapy, whether as standalone therapies or as supplements to conventional treatments. Licorice has diverse gastroprotective mechanisms. It can inhibit 11 beta-hydroxysteroid dehydrogenase (11β-HSD2), thereby boosting mineralocorticoid activity, and reducing inflammation. It might also carry out its effects via the suppression of phospholipase A2 and cyclooxygenase to reduce prostaglandin E2 and platelet aggregation [59]. Its antioxidants prevent ROS production and mitochondrial lipid peroxidation. Licorice also supports liver function by reducing serum enzymes and improving tissue pathology, enhancing overall gastrointestinal protection [60].

### 4.3. Probiotics and Prebiotics: Modulation of Gut Microbiota

Gastroesophageal reflux disease (GERD) is distinguished by acid reflux, mucosal inflammation, and gut microbial dysbiosis. Emerging research indicates that probiotics and prebiotics play an important role in regulating the gut flora to alleviate GERD symptoms. Probiotics are live microorganisms that provide health advantages when supplied in sufficient quantities, while prebiotics are non-digestible fibers that selectively promote beneficial bacteria. These endogenous agents play vital roles in maintaining gut homeostasis and gastrointestinal functions [61]. Probiotics are advantageous because they can help to eradicate *Helicobacter pylori* infections through various processes such as competitive inhibition, co-aggregation, enhanced mucus formation, bacteriocin secretion, and immunological modulation [62]. Their antagonistic effects differ depending on the *H. pylori* strain, and they can be used as adjuvant therapy, a drug delivery method, or immune system boosters in the treatment of *H. pylori* infections [62,63]. Due to their anti-inflammatory and antibacterial properties, probiotic strains such as Bifidobacterium and Lactobacillus can decrease the colonization of *Helicobacter pylori,* which could aggravate GERD [63]. They can also help to reduce esophageal hypersensitivity via enhancing gut–brain communication through the vagus nerve and improving the function of the mucosal barrier [64]. Prebiotics, such as inulin and fructooligosaccharides, preferentially stimulate the growth of good gut bacteria, resulting in short-chain fatty acid (SCFA) production. SCFAs such as butyrate have anti-inflammatory properties and improve epithelial integrity thereby making them able to reduce GERD-related mucosal damage [65]. Furthermore, the ability of prebiotics to control the metabolism of bile acid and lower bile reflux confer on these natural products a role in the management of GERD [66]. In a nutshell, combining the actions of probiotics and prebiotics is a promising strategy for GERD therapy. This strategy, known as synbiotics, may be able to restore microbial balance, reduce inflammation, and improve gut function. While additional research may be necessary to establish standardized dosages and strain-specific benefits of probiotics and prebiotics, the data for their application as an adjunctive therapy for GERD management are substantial [15].

### 4.4. Dietary, Physical and Physiological Aspects: Alkalizing and Anti-Reflux Regimens and Non-Drug Interventions

Gastroesophageal reflux disease (GERD) can be largely controlled with lifestyle adjustments, including dietary changes that reduce acid exposure to the esophagus. It is well-established that unhealthy eating practices, such as the consumption of heavy meals, eating late at night, and the consumption of caffeine and alcohol, can either exacerbate or trigger symptoms of GERD. Such practices can lead to increased acid production or relaxation of the lower esophageal sphincter (LES), consequently facilitating acid reflux [15,67,68]. Specific foods, including high-fat meals, alcohol, acidic beverages, and carbonated drinks are known to weaken esophageal sphincter pressure and worsen acid reflux [69]. In contrast, diets rich in fiber and low in acidity, like the Mediterranean diet, are capable of producing symptom alleviation similar to that of proton pump inhibitors [15,69]. Regulating meal size, timing, and macronutrient composition offers a cost-efficient alternative for pharmacological intervention in the management of GERD [15]. Alkalizing and anti-reflux diets discourage acidic food intake while promoting foods that neutralize gastric acid and reduce reflux episodes [15,70]. Alkalizing diets include foods with a neutral or alkaline effect on the body’s pH. Despite the stringent regulation of gastric pH, specific foods including bananas, melons, leafy greens, and cucumbers may assist in buffering stomach acidity and alleviating esophageal irritation [71]. Furthermore, alkaline water (pH > 8.0) has demonstrated the ability to denature pepsin, the enzyme implicated in mucosal damage associated with GERD, and offer symptomatic relief [71]. An anti-reflux diet, conversely, emphasizes the removal of dietary factors that can make the symptoms of GERD worse. Drinks and food items like caffeine, chocolate, high-fat meals, and acidic beverages that diminish lower esophageal sphincter pressure exacerbate acid reflux [69]. Dietary fibers from whole grains, fruits, and vegetables that facilitate stomach emptying and decrease transient esophageal sphincter relaxations can alleviate reflux episodes [5]. Incorporating alkalizing and anti-reflux food components may aid GERD patients in achieving symptom relief while reducing reliance on pharmacological treatments. In contrast, diets that are high in fermentable oligosaccharides, disaccharides, monosaccharides, and polyols (FODMAPs) can exacerbate digestive discomfort [16]. The implementation of a low-FODMAP diet has demonstrated potential in the relief of symptoms in individuals with functional gastrointestinal disorders. For example, in IBS-GERD patients, wheat noodle meals (high in FODMAP) resulted in a greater increase in TLESRs, GERD, and upper GI symptoms than rice noodle meals (low in FODMAP). This outcome may be attributed to the increased gas production by high FODMAP foods. In fact, adhering to a low FODMAP diet may bring relief from GERD symptoms in patients [16]. Beyond natural products and dietary interventions, non-drug, physical, and psychological approaches such as acupuncture, diaphragmatic breathing exercises, and relaxation techniques might be beneficial for GERD management alone or in conjunction with conventional therapies [72,73]. Acupuncture may help reduce symptoms by modulating esophageal motility and acid secretion [73]. Also, relaxation techniques like deep breathing and mindfulness may help manage GERD symptoms by reducing stress just as regular exercise aids in weight management and has the potential of lowering abdominal pressure that contributes to acid reflux [72,73,74].

## 5. Botanicals and Natural Products for the Management of GERD: Therapeutic Potential and Applications

Many natural and plant-based products have been used to treat GERD because of the antioxidant, anti-inflammatory, mucoprotective, and gastroprotective properties of their components (Table 1). Ginger, licorice, probiotics, slippery elm, Aloe vera, and melatonin, among others, enhance lower esophageal sphincter function, decrease gastric acid secretion, and protect the esophageal lining through various mechanisms. This section examines the therapeutic potential, mechanisms of action, and clinical evidence on the application of these products in the treatment of GERD.

### 5.1. Licorice Root

Licorice root, obtained from *Glycyrrhiza glabra* or *Glycyrrhiza uralensis*, has been historically applied to treat gastrointestinal disorders due to its demulcent properties [60]. The medical plant could increase mucus secretion and protect the esophagus lining against irritation. The therapeutic actions are believed to be facilitated by the principal active constituents—triterpenoid saponins and flavonoids [88]. Deglycyrrhizinated licorice (DGL) is usually the preferred choice for treatment because it has been established that excessive consumption of regular licorice might result in hypertension and hypokalemia [89].

Evidence from Preclinical and Clinical Studies: In many clinical trials, licorice proved to be effective in the treatment of gastrointestinal issues (Table 2). A randomized, placebo-controlled study conducted on an extract of *Glycyrrhiza glabra* (GutGard) showed that 30-day treatment with the extract significantly improved symptom severity and quality of life in patients with functional dyspepsia, a condition with some overlap in symptoms with GERD [90]. The deglycyrrhizinated licorice root extract could improve GERD symptoms and quality of life in individuals treated over 28 days according to another clinical study [91]. Also, when coupled with a triple treatment based on clarithromycin, licorice improved *H. pylori* eradication and promoted stomach healing, according to another randomized study conducted on 120 patients with dyspepsia, including those with prior peptic ulcers [92]. In terms of symptom relief, herbal formulations like DGL also performed better than antacids in a two-year observational trial of 58 GERD patients [93].

Mechanisms of Action: Licorice’s gastroprotective effects have been ascribed to its anti-inflammatory and mucosal-restorative properties. The natural compound can inhibit prostaglandin synthesis and lipoxygenase causing a reduction in gastric inflammation [58]. Licorice flavonoid (LF) is a key component of Glycyrrhizae Radix et Rhizoma, which could protect against gastric ulcers by enhancing gastric epithelial cell viability, reducing inflammation, and restoring the damaged mucosal barrier. It also promotes epithelial regeneration and angiogenesis by modulating the gut microbiota, increasing short-chain fatty acids, and upregulating mucus secretion through the EGFR/ERK pathway [104]. Consequently, it is a prospective treatment for GERD and other gastric disorders.

### 5.2. Traditional Chinese Medicine (TCM) Formulas

Traditional Chinese medicine (TCM) is popularly applied for the treatment of gastroesophageal reflux disease (GERD) through the use of a variety of herbal formulations. Medications such as Wu Zhu Yu Tang and Wendan Decoction could enhance esophageal motility, control stomach function, and reduce symptoms, thereby acting to provide an alternate or supplementary strategy to traditional acid-suppressing treatments [105,106]. Modified Xiaochaihu Decoction (MXD) reportedly demonstrated comparable efficacy to omeprazole for mild-to-moderate GERD and possibly aided in relapse prevention. Additionally, Sini Zuojin Decoction (SNZJD), a combination of Sini Powder (SNP) and Zuojin Pill (ZJP), has shown promise when used alongside traditional stomach medicines (SPTSM) [107].

Evidence from Preclinical and Clinical Studies: Research has shown that TCM is effective in treating GERD. Following a randomized clinical trial, for instance, MXD was found to significantly lower GERD-Q scores (*p* < 0.01) and improve esophageal motility, including lower esophageal sphincter pressure and swallowing function (*p* < 0.05) with lower relapse rates at 1-month (*p* < 0.01) and 3-month (*p* < 0.05) follow-ups [94]. Dai, et al. [4] conducted a network meta-analysis that ranked Jianpi therapy in combination with PPIs as the most effective in terms of clinical efficacy, while Ligan Hewei therapy was the most effective in terms of mucosal repair and symptom relief. This is a demonstration of the efficacy of both therapies. Finally, a meta-analysis of 13 studies with over 900 patient participants revealed that when SNZJD is combined with SPTSM, the improvement in symptoms and decrease in the recurrence and side effects were more evident than when given standard treatments alone [107].

Mechanisms of Action: TCM interventions combat the intricate pathogenesis of GERD such as anti-reflux barrier dysfunction, esophageal inflammation, hiatal hernia, transient lower esophageal sphincter relaxation (TLESR), and other psychological factors very well. Reports by Shih, et al. [106] indicated that Wu Zhu Yu Tang (Jianpi therapy) has anti-inflammatory, antioxidant, acid-suppressing, and mucosal-protective effects. Wendan Decoction (Ligan Hewei therapy) regulates orexin and leptin signaling [105] and modulates acid and bile secretion [108]. Acupuncture and acupoint therapy can aid in regulating the neuro-endocrine-immune system, improving esophageal sphincter pressure, and reducing acid reflux [109]. As shown previously, SNZJD’s mechanisms of action include bactericidal, acid-suppressive, antioxidant, and anti-inflammatory events. The PPI protein binding network indicates key targets such as JUN, IL6, and MAPK1. Li, et al. [107] show that SNZJD modulates various biological activities, including receptor activity and factor binding, via pathways such as TNF, estrogen, and AGE-RAGE signaling, as demonstrated by GO and KEGG studies. Research by Hsu, et al. [110] indicates a correlation between GERD and HBV, especially among women [111].

### 5.3. Chamomile

Chamomile is a plant belonging to the Asteraceae family that is widely used in traditional medicine to treat gastrointestinal disorders like GERD. Roman chamomile (*Chamaemelum nobile*) and German chamomile (*Chamomilla recutita*) are the two primary varieties of the plant. They both contain bioactive compounds that are essentially similar [53]. The dried flowers of chamomile are high in terpenoids and flavonoids, such as apigenin, quercetin, patuletin, chamazulene, and bisabolol, responsible for the herb’s anti-inflammatory, antispasmodic, and digestive relaxant properties [112,113].

Evidence from Clinical and Preclinical Studies: Chamomile has been shown to have the potential to alleviate symptoms of GERD when consumed after meals or just before bedtime. It acts as a digestive relaxant and could alleviate symptoms such as dyspepsia, flatulence, and stomach cramping [53,114]. Furthermore, chamomile could alleviate nervous excitability and regulate digestive function which makes it a prospective treatment for GERD-related discomfort [15]. The tea of chamomile has also been identified as a functional substance with ameliorative effects on GERD. It can reduce inflammation and stress-related acid reflux [15]. There are no clinical studies that explicitly focused on the treatment of GERD or dyspepsia with chamomile tea.

Mechanisms of Action: Chamomile’s gastroprotective properties are through multiple mechanisms. Essential oils from the plant have demonstrated inhibition of pro-inflammatory cytokines and oxidative stress. These include chamazulene, bisabolol oxides A and B, and α-bisabolol [53,112]. In addition, the herb contains mucilage, glycosides, and hydroxycoumarins which help to protect the mucosa and soothe the gastrointestinal mucosa. Chamomile’s spasmolytic effect on smooth muscles reduces acid reflux episodes by alleviating esophageal spasms and promoting gastric emptying. Apigenin, one of its primary bioactive constituents, possesses antioxidant and anti-inflammatory properties that may further improve mucosal protection [53].

### 5.4. Ginger

Ginger (*Zingiber officinale*) is an herb that is extensively used in both medicine and cuisine and is known for its digestive properties. The herb is usually sold in various forms, such as fresh and dry root, tea, capsules, and extracts. Bioactive compounds in ginger, including shogaols and gingerols, may play important roles in the anti-inflammatory, prokinetic, and gastroprotective effects [58,85,115,116].

Evidence from Preclinical and Clinical Studies: There are scientific reports on the therapeutic potential of ginger in the treatment of upper gastrointestinal (GI) disorders like functional dyspepsia (FD) and GERD. According to Aregawi, et al. [115], a randomized clinical trial reported a substantial improvement in GERD symptoms like postprandial fullness, early satiety, and epigastric pain when 1080 mg/day of ginger was administered for a period of four weeks. The efficacy of this herb in managing nausea, including pregnancy-induced nausea and vomiting (PINV), has also been emphasized in systematic reviews in which a daily dose of 1500 mg was shown to provide symptomatic relief [117]. It should be noted, however, that the results of FD treatment were not consistent possibly due to differences in dosage, extract formulations, and study methodologies [85,118], and randomized clinical trials focusing specifically on ginger extract for the treatment of GERD are scarce. Furthermore, ginger has demonstrated potential in enhancing the swallowing function of elderly individuals with dysphagia by increasing salivary substance P (SP) levels, a critical factor in the swallowing reflex, which may contribute to the prevention of aspiration pneumonia [119].

Mechanisms of Action: Ginger exhibits a therapeutic effect on GERD and other gastrointestinal disorders because of its impacts on gastrointestinal motility, inflammation, and neurotransmitter function. The plant also exhibits prokinetic effects by activating cholinergic pathways and causing spasmogenic activity. This leads to better gastroduodenal motility and gastric emptying [57,58]. The culinary herb improves gastrointestinal function and alleviates nausea by blocking intestinal cholinergic M3 and serotonergic 5-HT3 receptors (Palatty et al., 2013 [120]; Mohd Yusof, 2016 [121]; Schulz et al., 2022 [85]). Moreover, ginger exhibits anti-inflammatory and antioxidant characteristics. It can regulate the Nrf2 signaling pathway, inhibit p38 MAPK and NF-κB activity, and reduce oxidative stress and inflammation in the gastrointestinal tract (Farzaei et al., 2015 [39]; Samota et al., 2024 [116]). (Samota et al., 2024 [116]) indicated that gingerol, the principal bioactive ingredient, may demonstrate anti-tumor properties by suppressing glycolysis in gastric cancer cells, facilitating the formation of short-chain fatty acids, and strengthening the integrity of the gastrointestinal barrier.

### 5.5. Marshmallow Root (Althaea officinalis)

*Althaea officinalis* L., also known as marshmallow, is a medicinal plant belonging to the Malvaceae family. The anti-inflammatory, antioxidant, immunomodulatory, and mucoprotective effects are well-documented in the scientific literature [122]. The plant is typically used as a tea or supplement to treat gastrointestinal issues, such as GERD and stomach ulcers. The root contains bioadhesive and mucilaginous polysaccharides that produce a protective barrier across mucosal surfaces, thereby reducing inflammation and promoting tissue regeneration [75].

Evidence from Preclinical and Clinical Studies: Although a few studies show that *A. officinalis* has gastroprotective properties, clinical and preclinical evidence of its efficacy in GERD treatment is scarce. An in vitro study shows that it improves epithelial cell survival, adhesion, and extracellular matrix production, which supports its traditional usage in relieving mucosal irritation [123]. Research with experimental animal models has reported that it is as effective as omeprazole and misoprostol at lowering ulcer index, stomach acid, pepsin production, and oxidative stress [124]. Furthermore, *A. officinal* is extracts improve immunological function and macrophage activity, which aids mucosal healing [75].

Mechanisms of Action: The therapeutic effects of *A. officinalis* are largely due to its high polysaccharide content, which forms a mucin-like protective layer over inflamed mucosa and potentially minimizes esophageal damage in GERD [123]. The bioactive components of marshmallow, including flavonoids and antioxidants, influence inflammatory pathways by decreasing TNF-α and IL-6 while increasing cytoprotective enzyme activity [124]. Furthermore, its nitric oxide-mediated actions might aid stomach mucosal defense (Hage-Sleiman et al., 2011 [125]).

### 5.6. Slippery Elm (Ulmus rubra)

Slippery elm, which is derived from the inner bark of *Ulmus rubra,* has a long history of use in traditional medicine. The plant is indigenous to North America and has been used for soothing gastrointestinal and esophageal irritation for a long time. The mucilage content of *Ulmus rubra* (slippery elm) absorbs water and forms a viscous gel that constitutes a protective barrier over mucosal surfaces. The primary bioactive component of *Ulmus rubra*, mucilage, consists of high-molecular-weight polysaccharides, including pentose, hexose sugars, and uronic acids [126,127]. *Ulmus rubra* mucilage is available in lozenges, teas, and supplements as an herbal remedy for gastrointestinal disorders [76].

Evidence from Preclinical and Clinical Studies: Slippery elm has been investigated as part of the NC Gut Relief Formula, which demonstrated significant improvements in GERD-related symptoms, such as heartburn and indigestion, in a 16-week clinical study. Participants showed a 60–80% decrease in gastrointestinal pain, enhanced gut microbiota, and decreased intestinal permeability [128]. Almost fifty percent of the trial participants diminished their dependence on proton pump inhibitors, and numerous individuals successfully reintroduced trigger foods without experiencing symptom recurrence [128]. Notwithstanding these findings, investigations into the specific effects of slippery elm on GERD are scarce, as it is frequently used in multi-ingredient formulations instead of being examined independently [85].

Mechanisms of Action: The medicinal properties of slippery elm are primarily ascribed to its mucilage content, which creates a protective barrier across mucosal surfaces, safeguarding the esophagus from acid-related irritation and inflammation [76]. This gel-like mucilage may also promote the secretion of mucus and improve the integrity of the gastrointestinal lining. Moreover, the effects of slippery elm are linked to antioxidant, antimicrobial, and wound-healing properties. Every one of these qualities can help to maximize the possible benefits of this natural substance for GERD treatment [127].

### 5.7. Aloe Vera (Aloe barbadensis Miller)

Aloe vera (*Aloe barbadensis* Miller) is a medicinal plant that is widely used for its therapeutic benefits, especially in gastrointestinal health. Traditionally regarded as a “miracle gift of nature” [129], it has been incorporated into Chinese herbal medicine and nutritional supplements [130]. Aloe vera gel, which is obtained from the plant’s inner leaf, contains active phytochemicals such as anthraquinones (aloin A, aloin B, and aloe-emodin), polysaccharides, and flavonoids. These bioactives significantly contribute to the plant’s anti-inflammatory, antioxidant, and gastroprotective activities [131].

Evidence from Preclinical and Clinical Studies: Studies have shown that Aloe vera has considerable gastroprotective properties. Aloe vera may modulate the gut–brain axis by regulating gastric acid output and duodenal water content [132]. Clinical trials reveal that Aloe vera is efficient in treating GERD. Aloe vera syrup has been shown to improve GERD symptoms in a comparable manner to conventional drugs such as omeprazole and ranitidine without causing side effects [95,130,133]. In a clinical trial conducted over four weeks (Table 2), Aloe vera syrup was well-tolerated and proved effective at reducing GERD symptoms such as heartburn, regurgitation, and nausea [95]. People are now more aware of the therapeutic potential of Aloe vera in GERD, as indicated by a Howard University study in which 77.5% of pharmacy students were positive about its use for GERD control [134]. Aside from GERD, Aloe vera has therapeutic benefits for irritable bowel syndrome (IBS) and inflammatory bowel disease (IBD) like ulcerative colitis (UC) and Crohn’s disease. Aloe vera gel (200 mL/day) has been demonstrated in clinical trials to dramatically reduce disease activity ratings in UC patients without causing serious adverse effects [135]. Additionally, Aloe vera-derived nanovesicles and polysaccharides were observed to improve intestinal barrier integrity and reduce colitis-related inflammation [136,137].

Mechanism of Action: Aloe vera’s gastroprotective benefits stem from its ability to lower stomach acid secretion and inflammation while improving mucosal defense. Its anti-inflammatory properties are due to the suppression of pro-inflammatory mediators and oxidative stress [138]. Aloe vera regulates stomach acid secretion and protects against acid-induced damage [132]. Furthermore, Aloe vera polysaccharides could promote intestinal barrier strengthening and reduce inflammation in colitis models via the Nrf2/mitochondria axis [136].

### 5.8. Melatonin

Melatonin is an endogenous hormone produced from tryptophan in the brain. It has a range of physiological effects, the chief one of which is to regulate circadian rhythms. This highly lipophilic molecule scavenges free radicals and activates antioxidant enzymes, which helps to protect cells [139]. Although melatonin is mainly produced in the pineal gland, it is also produced in substantially higher amounts in the gastrointestinal system. In the gut, the hormone is involved in homeostasis, oxidative stress reduction, and modulation of inflammation [140].

Evidence from Clinical and Preclinical Studies: Melatonin has been shown in preclinical and clinical research to be effective in the treatment of GERD. It strengthens the lower esophageal sphincter (LES) and enhances gastrin release, lowering gastric acid secretion and reducing reflux episodes [77,78]. Clinical investigations show that a daily dose of 3 mg of melatonin relieves GERD symptoms in a manner comparable to that produced by 20 mg of the conventional PPI, omeprazole [77]. Furthermore, melatonin significantly improved GERD-related quality of life in comparison to both placebo and nortriptyline. According to [96], the sleep hormone could afford a safe and effective option for the management of functional heartburn. A more recent study reported that adding sublingual melatonin (3 mg/day) to omeprazole significantly improved GERD symptoms and quality of life compared to omeprazole alone, without increasing adverse events [78]. Apart from GERD, melatonin also has therapeutic benefits in managing gastrointestinal ulcers. A study on Helicobacter pylori-infected individuals reported that giving 5 mg of melatonin twice a day for 21 days decreased ulcer formation. This was taken as a protective impact of the compound on the stomach mucosa [141]. Furthermore, melatonin was successfully used to treat gastric/duodenal ulcers and regulate gastric acid secretion thanks to its antioxidative and anti-inflammatory properties [142].

Mechanisms of Action: Melatonin’s gastroprotective properties occur through a variety of mechanisms. The sleep hormone could prevent acid reflux and promote mucosal integrity by increasing the contractility of the LES [77]. Furthermore, melatonin regulates gastric acid secretion by upregulating gastrin levels and concurrently decreasing oxidative stress and inflammation in the gastrointestinal tract [140]. Its capacity to defend against Barrett’s esophagus and combat reflux esophagitis suggests a potential role in the long-term prevention of GERD [139].

### 5.9. Myrtus communis and Cydonia oblonga (Quince)

*Myrtus communis* or myrtle is a Mediterranean shrub belonging to the Myrtaceae family and has long been recognized for its medicinal benefits particularly in gastrointestinal health [143,144]. *Cydonia oblonga*, or quince, is a fruit used in traditional medicine for treating gastrointestinal disorders, with extracts such as quince sauce (QS) demonstrating significant gastroprotective effects [145].

Evidence from Preclinical and Clinical Studies: Myrtle has shown anti-inflammatory and antimicrobial properties that are beneficial for GERD and gastric ulcers. A study revealed that myrtle berry seed extract was able to offer protection against esophageal reflux-induced mucosal damage in rats by enhancing antioxidant enzymes [79]. According to [97], *Myrtus communis* extract was as effective as omeprazole in reducing GERD symptoms over six weeks in the participants of a clinical trial (IRCT2012072710410N1). In a study conducted by ref. [98], the combination of myrtle fruit syrup with omeprazole did not cause more improvement in GERD symptoms versus when the conventional drug was taken alone. However, post-treatment symptoms increased in the placebo group, indicating a potential role of the syrup in maintaining symptoms. Quince has also shown effectiveness in treating symptoms related to GERD; quince syrup was found to relieve symptoms like vomiting and stomach pain for pediatric patients better than ranitidine [80]. In addition, quince aqueous and hydroalcoholic extracts demonstrated effectiveness in reducing gastric acidity and ulcer severity in rats [146].

Mechanisms of Action: The gastroprotective benefits of myrtle are dependent on its antibacterial, anti-inflammatory, and antioxidant qualities, which help to combat harmful bacteria like *Helicobacter pylori*, alleviate inflammation, and protect the gastrointestinal lining. The exact mechanism underlying myrtle’s beneficial role against GERD has yet to be fully understood. Similarly, quince could aid the gastric mucosal defense by inhibiting harmful factors like gastric acidity and pepsin, promoting healing in gastrointestinal tissues, and modulating lower esophageal sphincter function [80,145].

### 5.10. Lonicerae (Chinese Honeysuckle Flower)

*Lonicerae* (Chinese Honeysuckle Flower), specifically Flos *Lonicerae* Japonicae, is a commonly used herb in traditional Chinese medicine that is known for its heat-clearing and detoxifying properties. The medicinal herb has popular ethnomedicinal applications for managing gastrointestinal (GI) disorders, just as it has more recently gained attention for its potential in treating gastroesophageal reflux disease (GERD) through its prokinetic and antioxidant effects. The standardized extract, GC-7101, is a prominent derivative studied for its therapeutic potential in GERD treatment [81].

Evidence from Preclinical and Clinical Studies: Studies indicate that GC-7101 effectively enhances esophageal lesions and mucosal thickness in animal models. It also increases glutathione levels and lowers myeloperoxidase activity. It restored lower esophageal sphincter (LES) tone, enhanced gastric emptying (GE), and improved gastrointestinal transit (GIT) more effectively than conventional prokinetics such as domperidone and mosapride [81]. In a rat model of reflux esophagitis (RE), Flos Lonicerae (LF) decreased esophageal and gastric mucosal lesions, lipid peroxidation, and collagen accumulation, and enhanced antioxidant indicators such as SOD, CAT, and GSH just like α-tocopherol [147]. Clinical trials provide more evidence of its efficacy. A double-blind, randomized research with 92 participants showed that individuals treated with GCWB104 (Flos Lonicerae extract) reported significant relief of symptoms like borborygmi, diarrhea, and fecal urgency, as well as improved Gastrointestinal Symptom Rating Scale (GSRS) ratings compared to those placed on a placebo [148].

Mechanisms of Action: The prokinetic and antioxidant properties of Lonicerae, namely GC-7101, have been implicated for its therapeutic benefits based on the findings of many preclinical studies. GC-7101 increased the formation of gastrointestinal mucus, increased PGE2 and NO levels, and controlled oxidative stress indicators, including NF-κB translocation and myeloperoxidase activity. The extract also enhanced the activities of antioxidant enzymes, including glutathione, superoxide dismutase (SOD), and catalase (CAT). This shows the contribution of antioxidant and anti-inflammatory properties of the herb to its ulcer-healing activity [149], and highlights its potential as an innovative prokinetic agent in the management of GERD [81].

### 5.11. STW5 (Iberogast)

STW5 (Iberogast) is a multi-herbal formulation with extracts from nine medicinal plants. These herbs are fresh plant extracts of bitter candytuft (Iberis amara) and dried extracts of eight other herbs namely, angelica root, chamomile flower, caraway fruit, St. Mary’s thistle fruit, balm leaves, peppermint leaves, greater celandine, and licorice root. The formulation is prepared using an alcohol-based extraction process at a fixed ratio, and this process might enhance the therapeutic effectiveness of the formulation in addressing GERD and functional gastrointestinal disorders (FGIDs) [82].

Evidence from Clinical and Preclinical Studies: STW5’s effects on gastrointestinal motility have been reported in prior studies. In one experiment, guinea pig stomach fundus muscles were relaxed while antral contractions increased following treatment with the herbal formulation [82]. Human studies indicated that STW5 increases proximal gastric volume and antral pressure waves while having no effect on pyloric or duodenal pressures or gastric emptying [150]. A double-blind, randomized, placebo-controlled experiment found that STW5 could reduce “GERD” and “regurgitation” subscale scores in patients with functional dyspepsia and reflux symptoms [151]. Furthermore, in GERD patients, STW5 decreased acidic reflux occurrences while increasing the time to acid awareness in reflux esophagitis (*p* = 0.042). STW5 was reported to be effective for pediatric patients with GERD, reducing symptom severity by up to 76% [152].

Mechanisms of Action: STW5 directly affects gastrointestinal motility by acting on smooth muscle, independent of neural connections. It relaxes fundus muscles and increases antral contractions, and it exhibits spasmolytic properties by decreasing acetylcholine- and histamine-induced contractions while boosting baseline tone [82]. STW5 modulates intestinal slow wave amplitude and frequency through receptors such as 5-HT4, 5-HT3, M3, and opioid receptors, suggesting a role in visceral sensation modulation. Beyond motility, STW5 also has protective effects on the gastric mucosa, reducing acid hypersecretion and promoting mucin and prostaglandin E2 release [153]. Finally, the anti-inflammatory properties of the formulation and its ability to regulate intestinal secretion also make it beneficial for some gastrointestinal disorders including constipation [154].

### 5.12. Raft-Forming Agents (Alginate, Pectin, Carbenoxolone)

Raft-forming agents, such as alginate, pectin, and carbenoxolone, afford a kind of non-systemic treatment strategy to treat GERD by creating protective barriers that inhibit acid reflux. Alginate is a natural polymer derived from algae that serves as the primary component in many formulations. Pectins are plant-derived polysaccharides that function as binding agents in cell walls. Carbenoxolone is a glycyrrhizinic acid derivative sourced from licorice that enhances raft stability and enhances mucosal protection [155].

Evidence from Preclinical and Clinical Studies: Clinical studies validate the effectiveness of alginate-based formulations in the treatment of GERD (Table 2). In contrast to antacids, alginates produce a gel when exposed to gastric acid, resulting in a floating foam barrier that diminishes reflux episodes [83]. A meta-analysis of 14 trials (*N* = 2095) revealed that alginates were significantly more effective than placebo and antacids (OR: 4.42), while they were less effective than PPIs or H2Ras [84]. According to a clinical trial (EudraCT 2012-002188-84) [99], Gaviscon Double Action, an alginate-based formulation, causes improvement in reflux symptoms when compared to placebo and appeared safe in participants. A similar product, Gaviscon Advance, significantly reduced GERD symptoms in PPI-refractory patients over seven days in another study (EudraCT 2011-005486-21) [100]. However, a study [101] reported that adding non-bicarbonate alginate (Lamina G) to PPIs was not better than PPIs alone for the control of GERD symptoms. Raft-forming drugs have demonstrated advantages in the management of GERD in both pregnant and pediatric populations due to their non-systemic properties which render them appropriate for these specific clinical groups (Quartarone, 2013 [156]).

Mechanisms of Action: Upon exposure to gastric acid, alginate and pectin make a gel-like matrix that, along with carbon dioxide generated by bicarbonate, creates a buoyant raft that displaces the postprandial acid pocket. These rafts generally prevent acid from reaching the esophagus, thereby offering mechanical protection against reflux [83]. Carbenoxolone, with a steroidal structure, strengthens mucosal resistance by stabilizing the raft and improving barrier function. These agents function autonomously from acid suppression.

### 5.13. D-Limonene

D-limonene is a monocyclic monoterpene found in citrus peel with potential in managing GERD symptoms without altering gastric acid levels. The terpenoid may serve as a natural alternative for heartburn relief because it can preserve digestive function and maintain normal peristalsis. Since D-limonene could neutralize gastric acid and enhance mucosal defense, it can be regarded as a viable candidate for GERD treatment [85].

Evidence from Preclinical and Clinical Studies: Two studies under a U.S. patent demonstrated significant symptom remission rates in patients with GERD and persistent heartburn following the administration of D-limonene [85] but extensive clinical trials are still needed for validation. A few other studies found that D-limonene has gastroprotective effects that are unrelated to GERD. For instance, D-limonene protects against ethanol-induced gastric ulcers by enhancing mucus secretion, reducing oxidative stress, and modulating inflammation. It has also been shown in animal models to reduce pro-inflammatory cytokines (TNF-α, IL-6, and IL-1β) and elevate anti-inflammatory IL-10 levels [86]. Additionally, it alleviated NSAID-induced stomach injury without affecting acid secretion, thereby advancing its gastroprotective properties [50]. These findings support the therapeutic efficiency of D-limonene for GERD and related gastrointestinal disorders.

Mechanisms of Action: D-limonene may afford gastroprotection by strengthening the intestinal epithelial barrier. Its function includes enhancing transepithelial electrical resistance (TEER) and diminishing paracellular permeability via upregulating tight junction proteins (occludin, claudin-1, and ZO-1) and an adherens junction protein (E-cadherin) [157]. D-limonene also acts as a cannabinoid receptor type-1 (CB1R) antagonist by mimicking pharmacological inhibitors to promote intestinal barrier restoration.

### 5.14. Artemisia asiatica

*Artemisia asiatica* Nakai (Compositae) is a perennial herb that has significant ethnomedicinal applications in Asia and some parts of Europe. Experimental formulations, such as DA-5204 (Stillen 2X) and DA-9601 (StillenTM), utilize ethanol extracts of *A. asiatica* to enhance stomach retention and cytoprotective qualities. These formulations may address gastritis and gastric ulcers through antioxidative processes. DA-5204, containing 90 mg of *A. asiatica* extract per tablet, is employed in the treatment of GERD, particularly for alleviating moderate esophageal irregularities [87].

Evidence from Preclinical and Clinical Studies: Preclinical investigations indicate *A. asiatica*’s efficacy in alleviating reflux esophagitis and preventing its onset in animal models [158,159]. In experimental rats, the ethanol extract of *A. asiatica* at dosages of 30 mg/kg or 100 mg/kg showed superior efficacy in preventing esophageal erosion compared to ranitidine [160]. Oxidative stress, a critical element in GERD-associated disorders such as Barrett’s esophagus and esophageal cancer, was substantially reduced by the antioxidative capabilities of *A. asiatica* [161]. According to a study [87], DA-5204 (*Artemisia asiatica* extract) combined with a PPI significantly reduced minimal esophageal mucosal changes, although there was no improvement in endoscopic healing [87]. Similarly, DA-9601 exhibited enhanced efficacy in the treatment of erosive gastritis relative to cetraxate, and this was accompanied by a favorable safety profile [162].

Mechanisms of Action: The medicinal effects of *A. asiatica* are ascribed to its strong antioxidative and anti-inflammatory capabilities. The extract mitigates oxidative stress-induced esophageal ulceration and functions as a viable adjunct to acid suppression therapy [160]. *A. asiatica* also demonstrates anti-inflammatory effects by inhibiting NF-κB and AP-1 transcriptional activity and decreasing the synthesis of nitric oxide (NO), tumor necrosis factor (TNF)-α, and prostaglandin E2 (PGE2) in macrophages [163]. The effects are mediated by the suppression of Src/Syk for NF-κB translocation and TRAF6/JNK for AP-1 activation [163]. One of the principal bioactive components responsible for these anti-inflammatory processes is luteolin, a flavonoid found in *A. asiatica* [161].

### 5.15. Phenolics and Flavonoids: Curcumin, Quercetin, and Vitamin E

**Figure 1:**
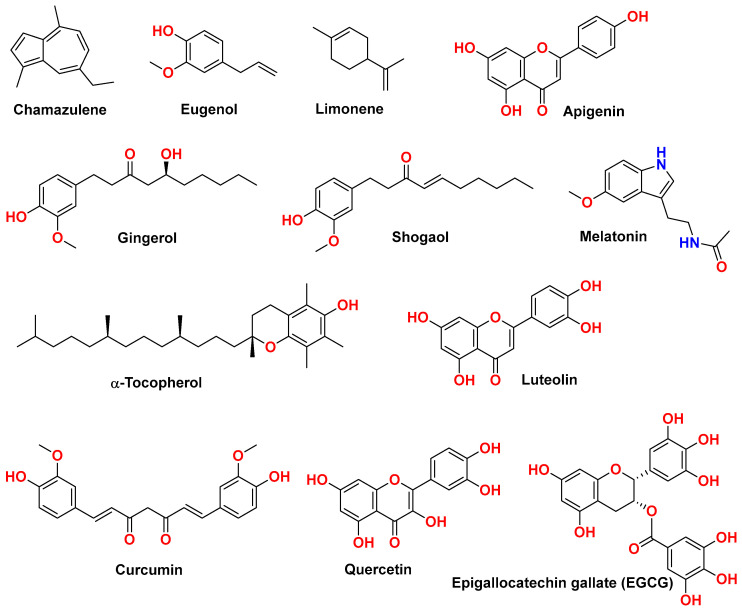
Structures of selected active compounds from botanicals and natural agents used in treating GERD and its symptoms.

Phenolic substances like curcumin, quercetin, and vitamin E (α-tocopherol, Figure 1) are well known for their antioxidants and anti-inflammatory properties. The flavonoid, curcumin, is found in Curcuma longa (turmeric) and has strong antioxidant and anti-inflammatory effects [42]. Quercetin is a flavonoid abundant in numerous fruits and vegetables and is highly rated for both antioxidant and anti-inflammatory potentials [164]. The compound is found in the plants Euphorbia hirta and Rumex aquaticus as quercetin-3-O-β-D-glucuronopyranoside (QGC), which has displayed gastroprotective effects [165]. Vitamin E, or α-tocopherol, is a lipid-soluble antioxidant known for its strong potential to neutralize reactive oxygen species (ROS) and boost mucosal defense systems [40].

Evidence from Preclinical and Clinical Studies: Preclinical studies demonstrate the protective effects of these antioxidants in experimental GERD models. Quercetin (100 mg/kg) and α-tocopherol (16 mg/kg) both reduced the severity of esophagitis in rats by reducing acid secretion, membrane lipid peroxidation, and plasma histamine while enhancing antioxidant enzyme activity [40]. Similarly, QGC from *Rumex aquaticus* inhibited reflux esophagitis and gastritis by reducing ulcer index, gastric volume, and acid output. It also performed better than both quercetin and omeprazole in increasing gastric pH and antioxidant defenses [165]. The anti-reflux effect of curcumin was demonstrated when it was discovered to prevent acute reflux esophagitis and mitigate mixed reflux esophagitis to an even greater extent than lansoprazole [166]. In addition, an extract of *Curcumae longae* Rhizoma reduced oxidative stress and inflammation in rats surgically induced with reflux esophagitis by inhibiting the synthesis of pro-inflammatory proteins mediated by NF-κB [167]. Curcumin improved acute corrosive esophagitis caused by sodium hydroxide in a dose-dependent manner and exhibited considerable protection of the mucosal and muscularis layers at a dosage of 200 mg/kg [42]. The extract of *Euphorbia hirta* and its flavonoids (kaempferol, quercetin, and rutin) provide supplementary corroborative evidence. Analogous to omeprazole, they demonstrated gastroprotective effects in GERD-induced animals by enhancing gastric mucus secretion, diminishing histamine and H⁺/K⁺-ATPase levels, and fortifying antioxidant defenses [48].

Mechanisms of Action: The therapeutic effects of curcumin, quercetin, and vitamin E are carried out through their antioxidant, anti-inflammatory, and cytoprotective mechanisms. These natural antioxidants can easily mop up reactive oxygen species (ROS) which are important for the development of mucosal damage in GERD. Inhibiting NF-κB signaling reduces pro-inflammatory cytokines such as TNF-α, interleukins, and prostaglandins, leading to their anti-inflammatory effects (Lee et al., 2021 [167]). Quercetin and QGC reduce lipid peroxidation markers such as malondialdehyde, increase gastric pH, and decrease acid secretion, thereby mitigating gastric and esophageal injury (Min et al., 2009 [165]). Mitigation of esophageal injury and promotion of mucosal healing by curcumin occur through the modulation of oxidative stress-induced inflammatory pathways [166]. As for vitamin E, there is evidence that it protects the esophageal mucosa from oxidative damage through its lipid-soluble antioxidant property, which keeps cell membranes stable [40].

### 5.16. Probiotics and Prebiotics

Both probiotics and prebiotics can function together to strengthen the immune system, reduce inflammation, and improve digestive health. Their activities may also be harnessed to alleviate gastrointestinal disorders such as irritable bowel syndrome and GERD [134]. *Bifidobacterium bifidum*, *Lactobacillus johnsonii*, and *Lactobacillus gasseri* have been identified as some of the specific probiotic strains that could provide targeted benefits like improved digestion, enhanced mucosal protection, and reduced gastric acid production.

Evidence from Preclinical and Clinical Studies: Recent research is revealing the importance of gut microbiome dysbiosis in the development of GERD, more so as distinct microbial compositions have now been observed in reflux esophagitis (RE) and non-erosive reflux disease (NERD) [36]. Lipopolysaccharides (LPS) produced by Gram-negative bacteria reportedly activated TLR-4-mediated inflammatory pathways consequently producing altered gastric motility and dysfunction of the lower esophageal sphincter (LES) [168,169]. Dysbiosis is further exacerbated by dietary factors, particularly high-fat ingestion, which affects microbial metabolism and increases the risk of GERD [170,171]. Clinical analyses have confirmed that probiotic supplementation can enhance gastric emptying, reduce acid reflux episodes, and ultimately improve gastrointestinal motility. In 2020, a systematic study reported that probiotic use caused a 79% improvement in GERD symptoms, including dyspepsia, regurgitation, and indigestion, including a 40% reduction in reflux episodes [17].

Mechanisms of Action: Prebiotics and probiotics improve GERD symptoms by changing the gastrointestinal flora in the gut to reestablish microbial equilibrium and reduce inflammatory reactions. According to a report, Lactobacillus johnsonii decreases gastric acid production, Bifidobacterium bifidum increases mucous secretion to improve gastric protection, and *Lactobacillus gasseri* facilitates digestion. These beneficial microbes enhance LES function and reduce reflux severity by inhibiting pro-inflammatory pathways. They can achieve this by alleviating oxidative stress and influencing gastric motility through bacterial glucose metabolism [17].

## 6. Challenges, Limitations, and the Path Forward in Using Natural Products for GERD

Without a doubt, natural products have potential therapeutic benefits for GERD, but their clinical application still faces several challenges just like other botanicals and natural remedies used in treating many other pathological conditions. Major barriers to using various natural products in the management of GERD include quality control concerns, unethical production practices in certain places, a lack of compositional data, and insufficient safety information, especially concerning herb-drug interactions and adverse effects. Additionally, inconsistent clinical evidence, research limitations, and regulatory gaps further hinder their acceptance [172]. Overcoming these issues necessitates thorough research, standardization, and improved regulatory control to guarantee the efficacy, safety, and incorporation of these natural products into conventional healthcare.

Safety Issues: Ensuring the safety of natural products used for pharmacological purposes is crucial, as unintended interactions with endogenous pathways can lead to adverse effects. For example, even though licorice has therapeutic values in the relief of GERD symptoms, it was found to contain glycyrrhizin, which can cause hypertension and electrolyte imbalances, necessitating the use of deglycyrrhizinated licorice (DGL) for safer consumption [89]. In the same vein, chamomile has a propensity to induce allergic reactions in individuals who are allergic to Asteraceae plants [173], whereas high doses of ginger (>5 g/day) can result in gastrointestinal discomfort and risk to those with bleeding disorders [58,117]. Pregnant women and infants are usually advised to use fennel seed oil with caution because it may induce skin reactions, just as the association of D-limonene with allergic reactions and nausea has been reported. Furthermore, the cytoprotective mechanisms of Aloe vera, for example, need to be clarified, and standardized formulations generated, even though the potential therapeutic qualities are widely documented [132].

Inconsistent Clinical Evidence: There is often a dearth of large-scale, well-designed clinical trials to prove the efficacy of natural products, and this is one key issue limiting their use in GERD. For instance, traditional Chinese medicine (TCM) reportedly faced issues with intermediate trial quality, selection bias, and missing data that resulted in poor overall quality of evidence [107]. Similarly, research on botanicals including marshmallow root, slippery elm, *Myrtus communis*, and *Cydonia oblonga* is limited, which reduced their usefulness for GERD treatments. The clinical effectiveness of probiotics and prebiotics in the management of GERD also varies, and this is why it is important to employ strain-specific studies to assess their therapeutic potential.

Methodological Challenges in Research: Variability in extract composition, dosing, and study design has resulted in inconsistent findings for botanicals such as ginger and Aloe vera, as well as polyphenols such as curcumin and quercetin. Further standardization in clinical trials is necessary to establish optimal dosages and therapeutic effectiveness [132]. Also, natural raft-forming substances like alginate and pectin that are used to treat mild to moderate GERD need to be further researched to make the formulations more stable and effective [84].

Regulatory Obstacles: Some herbal remedies used to treat GERD still face barriers from regulatory agencies. Several, including slippery elm and STW5, are not regulated by the FDA for safety or efficacy, raising concerns about product consistency, safety, and long-term effects [76,85,128]. The absence of standardized regulations results in variability in product quality, making it difficult to ensure their reliability in clinical practice.

## 7. Conclusions

Natural products are potentially effective for treating GERD through various mechanisms, including acid suppression, mucosal protection, anti-inflammatory activity, and gut microbiota modification, among others. The effectiveness of several bioactive substances like flavonoids, polyphenols, plant-derived oils, probiotics, and dietary components has been demonstrated in both clinical and preclinical studies. Despite their therapeutic potential, much remains to be done before most natural products can be fully incorporated into conventional GERD treatment. It is important that confounding factors such as variability in bioavailability, lack of dosage uniformity, and regulatory barriers be duly addressed before their applications in clinical settings. Additionally, while some natural products show comparable effectiveness to conventional treatments such as proton pump inhibitors, further well-designed clinical trials are needed to validate their long-term efficacy and safety. Overcoming these constraints through meticulous research, increased standardization, and regulatory advances will boost natural products’ acceptance as complementary or alternative treatments for GERD. Future research should center on improving formulations, determining precise therapeutic dosages, and investigating potential synergistic effects with existing pharmaceutical medicines. By resolving these gaps, natural products can be better integrated into GERD management, providing patients with safer and more comprehensive treatment alternatives.

## Figures and Tables

**Table 1:** Bioactive compounds and their therapeutic mechanisms in GERD.

Botanical/Extract/Product	Bioactive Compounds	Major Findings and Mechanisms	References
Flavonoids and Polyphenols (e.g., Quercetin, EGCG, Curcumin)	Quercetin, Epigallocatechin gallate (EGCG), Curcumin	Antioxidant, anti-inflammatory, mucosal protection, suppression of NF-κB activation, reduction of oxidative stress in GERD patients	[38,39,40,42]
*Syzygium aromaticum* (Clove) Essential Oil	Eugenol	Gastroprotective effects due to an increase in gastric mucus production rather than alterations in gastric juice volume, acidity, nitric oxide, or endogenous sulfhydryl activity	[49]
*Citrus aurantium* Essential Oil	Limonene	Protects gastric mucosa by enhancing mucus production and preserving basal prostaglandin E2 (PGE2) levels; does not affect acid secretion, or serum gastrin, making it a potential alternative to PPIs without rebound acid hypersecretion	[50]
Aloe vera	Polysaccharides, Flavonoids	Reduces GERD symptoms, promotes mucosal healing, anti-inflammatory actions	[54]
Licorice (*Glycyrrhiza glabra*)	Glycyrrhizin, Flavonoids	Enhances mucus secretion, gastroprotective, anti-inflammatory	[55,59]
Ginger (*Zingiber officinale*)	Gingerols, Shogaols	Prokinetic properties, reduces reflux incidents, mucosal protection	[57,58]
Chamomile (*Matricaria chamomilla*)	Apigenin, Chamazulene	Anti-inflammatory, mucosal healing	[53]
Probiotics & Prebiotics	Lactobacillus, Bifidobacterium	Modulate gut microbiota, reduce inflammation, enhance esophageal barrier function	[61,64]
Marshmallow Root (*Althaea officinalis*)	Polysaccharides, Flavonoids	Forms mucosal barrier, reduces esophageal irritation	[75]
Slippery Elm (Ulmus rubra)	Mucilage (polysaccharides)	Forms protective barrier, alleviates acid-induced irritation	[76]
Melatonin	Tryptophan-derived hormone	Strengthens lower esophageal sphincter (LES), reduces acid secretion, gastroprotective	[77,78]
*Myrtus communis *(Myrtle) and *Cydonia oblonga *(Quince)	Polyphenols, Antioxidants	Anti-inflammatory, protects against esophageal damage, reduces GERD symptoms	[79,80]
*Lonicerae *(Honeysuckle)	Polyphenols, Flavonoids	Prokinetic, reduces oxidative stress, improves LES function	[81]
STW5 (Iberogast)	Blend of 9 medicinal plants	Reduces reflux episodes, enhances gastric motility, mucosal protection	[82]
Raft-forming Agents (Alginate, Pectin, Carbenoxolone)	Alginate, Pectin, Glycyrrhizin derivatives	Forms protective raft, prevents acid reflux, mucosal healing	[83,84]
D-Limonene	Monoterpenes	Neutralizes gastric acid, protects mucosa, anti-inflammatory	[85,86]
*Artemisia asiatica*	Luteolin, Polyphenols	Anti-inflammatory, antioxidative, mucosal healing	[87]
Curcumin, Quercetin, Vitamin E	Curcumin, Quercetin, α-Tocopherol	Antioxidant, anti-inflammatory, reduces esophageal damage	[40,42]

**Table 2:** Randomized clinical trials investigating the effectiveness of natural products in the treatment of GERD.

Natural Compound/Product	Type of Clinical Trial	Number of Participants	Treatment Duration	Findings Relevant to GERD	References
GutGard^®^ (De-glycyrrhizinated licorice root extract)	Phase III, randomized, double-blind, placebo-controlled trial	200	28 days	Significantly better quality of life, earlier symptom resolution (heartburn, regurgitation), improvements starting as early as day 7	[91]
Modified Xiaochai-hu Decoction (MXD)	Randomized, double-blind, double-simulation controlled trial	288	4 weeks + 3-month follow-up	Significant symptom improvement, better esophageal motility, lower relapse rate at 1 and 3 months, comparable to omeprazole	[94]
Aloe vera syrup	Randomized controlled trial (pilot study)	79	4 weeks	Reduced frequency of GERD symptoms (heartburn, regurgitation, flatulence, belching, dysphagia, nausea, vomiting), well-tolerated, no adverse events leading to withdrawal	[95]
Melatonin	Randomized, placebo-controlled clinical trial	60	3 months	Significant improvement in GERD-related quality of life scores, fewer adverse events compared to nortriptyline and placebo	[96]
Sublingual melatonin	Randomized, double-blind clinical trial	78 (72 completed)	4 weeks	Greater improvements in heartburn, epigastric pain, GERD symptom scores (*p*-values: 0.04, 0.03, 0.0001), higher quality of life scores (*p* = 0.0001)	[78]
*Myrtus communis* L. (Myrtle) extract	Randomized, double-blind, controlled trial (IRCT2012072710410N1)	45	6 weeks	Significant symptom reduction in all groups, no significant differences between treatment groups	[97]
*Myrtus communis* L. fruit syrup	Randomized, double-blind clinical trial (IRCT2016061828521N1)	76 (children aged 1–7)	8 weeks + 4-week post-treatment	No significant difference in GERD symptom scores between groups, more stable symptoms in the myrtle group post-treatment, improved appetite (*p* = 0.018, *p* = 0.042)	[98]
Gaviscon Double Action (Alginate)	Randomized, double-blind, parallel-group trial (EudraCT 2012-002188-84)	110	7 days	Greater decrease in RDQ scores (*p* = 0.0033), better overall treatment evaluation (*p* = 0.0005), no significant differences in adverse events	[99]
Gaviscon Advance (Alginate)	Randomized, double-blind, placebo-controlled trial (EudraCT 2011-005486-21)	136	7 days	Greater reduction in reflux symptoms, fewer nights with symptoms, statistically significant improvements	[100]
Lamina G (Non-bicarbonate alginate)	Randomized clinical trial (CRIS KCT0002297)	120	Not specified	No significant difference in symptom resolution or quality of life compared to PPI alone	[101]
DA-5204 (Artemisia asiatica extract)	Randomized, double-blind, placebo-controlled trial	70	4 weeks	Similar endoscopic healing rates compared to PPI alone, significantly lower rate of residual minimal change (*p* < 0.001)	[87]
Mucosave® (Opuntia ficus-indica, Olea europaea extracts)	Randomized, double-blind, controlled trial	118	2 months	56.5% reduction in GERD-HRQoL scores, 59.1% reduction in GSAS scores, significant decrease in heartburn and acid regurgitation episodes (*p* < 0.01)	[102]
LiHuo probiotics	Randomized, double-blind, placebo-controlled trial	120	8 weeks treatment + 4 weeks maintenance	Study ongoing; aims to assess impact on gut microbiome and GERD symptoms during long-term PPI use	[103]

## Data Availability

Not applicable.

## References

[1] Antunes C., Aleem A., Curtis S.A. (2025). Gastroesophageal Reflux Disease.

[2] Vakil N., van Zanten S.V., Kahrilas P., Dent J., Jones R. (2006). The Montreal definition and classification of gastroesophageal reflux disease: A global evidence-based consensus. Am. J. Gastroenterol..

[3] Gawron A.J., French D.D., Pandolfino J.E., Howden C.W. (2014). Economic evaluations of gastroesophageal reflux disease medical management. PharmacoEconomics.

[4] Dai Y.K., Wu Y.B., Wen H., Li R.L., Chen W.J., Tang C., Lu L., Hu L. (2020). Different Traditional Herbal Medicines for the Treatment of Gastroesophageal Reflux Disease in Adults. Front. Pharmacol..

[5] El-Serag H.B., Sweet S., Winchester C.C., Dent J. (2014). Update on the epidemiology of gastro-oesophageal reflux disease: A systematic review. Gut.

[6] Kim Y.S., Kim N., Kim G.H. (2016). Sex and Gender Differences in Gastroesophageal Reflux Disease. J. Neurogastroenterol. Motil..

[7] Azer S.A., Hashmi M.F., Reddivari A.K.R. (2024). Gastroesophageal Reflux Disease (GERD).

[8] Durazzo M., Lupi G., Cicerchia F., Ferro A., Barutta F., Beccuti G., Gruden G., Pellicano R. (2020). Extra-Esophageal Presentation of Gastroesophageal Reflux Disease: 2020 Update. J. Clin. Med..

[9] Hom C., Vaezi M.F. (2013). Extra-esophageal manifestations of gastroesophageal reflux disease: Diagnosis and treatment. Drugs.

[10] De Giorgi F., Palmiero M., Esposito I., Mosca F., Cuomo R. (2006). Pathophysiology of gastro-oesophageal reflux disease. Acta Otorhinolaryngol. Ital..

[11] Mussbacher M., Salzmann M., Brostjan C., Hoesel B., Schoergenhofer C., Datler H., Hohensinner P., Basílio J., Petzelbauer P., Assinger A. (2019). Cell Type-Specific Roles of NF-κB Linking Inflammation and Thrombosis. Front. Immunol..

[12] Rettura F., Bronzini F., Campigotto M., Lambiase C., Pancetti A., Berti G., Marchi S., de Bortoli N., Zerbib F., Savarino E. (2021). Refractory Gastroesophageal Reflux Disease: A Management Update. Front. Med..

[13] Maideen N.M.P. (2023). Adverse Effects Associated with Long-Term Use of Proton Pump Inhibitors. Chonnam Med. J..

[14] Newberry C., Lynch K. (2019). The role of diet in the development and management of gastroesophageal reflux disease: Why we feel the burn. J. Thorac. Dis..

[15] Herdiana Y. (2023). Functional Food in Relation to Gastroesophageal Reflux Disease (GERD). Nutrients.

[16] Plaidum S., Patcharatrakul T., Promjampa W., Gonlachanvit S. (2022). The Effect of Fermentable, Oligosaccharides, Disaccharides, Monosaccharides, and Polyols (FODMAP) Meals on Transient Lower Esophageal Relaxations (TLESR) in Gastroesophageal Reflux Disease (GERD) Patients with Overlapping Irritable Bowel Syndrome (IBS). Nutrients.

[17] Cheng J., Ouwehand A.C. (2020). Gastroesophageal Reflux Disease and Probiotics: A Systematic Review. Nutrients.

[18] Savarino E., Zentilin P., Savarino V. (2013). NERD: An umbrella term including heterogeneous subpopulations. Nat. Rev. Gastroenterol. Hepatol..

[19] Hershcovici T., Fass R. (2010). Nonerosive Reflux Disease (NERD)—An Update. J. Neurogastroenterol. Motil..

[20] Sawada A., Sifrim D., Fujiwara Y. (2023). Esophageal Reflux Hypersensitivity: A Comprehensive Review. Gut Liver.

[21] Giacchino M., Savarino V., Savarino E. (2013). Distinction between patients with non-erosive reflux disease and functional heartburn. Ann. Gastroenterol..

[22] Sharma P., Yadlapati R. (2021). Pathophysiology and treatment options for gastroesophageal reflux disease: Looking beyond acid. Ann. N. Y. Acad. Sci..

[23] Muszyński D., Kudra A., Sobocki B.K., Folwarski M., Vitale E., Filetti V., Dudzic W., Kaźmierczak-Siedlecka K., Połom K. (2022). Esophageal cancer and bacterial part of gut microbiota—A multidisciplinary point of view. Front. Cell. Infect. Microbiol..

[24] Richter J.E. (2009). Role of the gastric refluxate in gastroesophageal reflux disease: Acid, weak acid and bile. Am. J. Med. Sci..

[25] Souza R.F., Huo X., Mittal V., Schuler C.M., Carmack S.W., Zhang H.Y., Zhang X., Yu C., Hormi-Carver K., Genta R.M. (2009). Gastroesophageal reflux might cause esophagitis through a cytokine-mediated mechanism rather than caustic acid injury. Gastroenterology.

[26] Tack J., Pandolfino J.E. (2018). Pathophysiology of Gastroesophageal Reflux Disease. Gastroenterology.

[27] Rieder F., Biancani P., Harnett K., Yerian L., Falk G.W. (2010). Inflammatory mediators in gastroesophageal reflux disease: Impact on esophageal motility, fibrosis, and carcinogenesis. Am. J. Physiol. Gastrointest. Liver Physiol..

[28] Huo X., Zhang X., Yu C., Zhang Q., Cheng E., Wang D.H., Pham T.H., Spechler S.J., Souza R.F. (2014). In oesophageal squamous cells exposed to acidic bile salt medium, omeprazole inhibits IL-8 expression through effects on nuclear factor-κB and activator protein-1. Gut.

[29] Gao T., Joyce B.T., Liu L., Zheng Y., Dai Q., Zhang Z., Zhang W., Shrubsole M.J., Tao M.H., Schwartz J. (2016). DNA methylation of oxidative stress genes and cancer risk in the Normative Aging Study. Am. J. Cancer Res..

[30] Han D., Zhang C. (2022). The Oxidative Damage and Inflammation Mechanisms in GERD-Induced Barrett’s Esophagus. Front. Cell Dev. Biol..

[31] Wang Q., Ma S., Liu M., Tao Y., Sun Z. (2025). Gut microbiota, inflammatory cytokines and gastro-esophageal reflux disease: A Mendelian randomization analysis. Medicine.

[32] Wang K., Wang S., Chen Y., Lu X., Wang D., Zhang Y., Pan W., Zhou C., Zou D. (2024). Causal relationship between gut microbiota and risk of gastroesophageal reflux disease: A genetic correlation and bidirectional Mendelian randomization study. Front. Immunol..

[33] Okereke I., Hamilton C., Wenholz A., Jala V., Giang T., Reynolds S., Miller A., Pyles R. (2019). Associations of the microbiome and esophageal disease. J. Thorac. Dis..

[34] Magne F., Gotteland M., Gauthier L., Zazueta A., Pesoa S., Navarrete P., Balamurugan R. (2020). The Firmicutes/Bacteroidetes Ratio: A Relevant Marker of Gut Dysbiosis in Obese Patients?. Nutrients.

[35] Liu Y., Yu J., Yang Y., Han B., Wang Q., Du S. (2024). Investigating the causal relationship of gut microbiota with GERD and BE: A bidirectional mendelian randomization. BMC Genom..

[36] D’Souza S.M., Houston K., Keenan L., Yoo B.S., Parekh P.J., Johnson D.A. (2021). Role of microbial dysbiosis in the pathogenesis of esophageal mucosal disease: A paradigm shift from acid to bacteria?. World J. Gastroenterol..

[37] Jiang Z., Wang J., Shen Z., Zhang Z., Wang S. (2021). Characterization of Esophageal Microbiota in Patients With Esophagitis and Esophageal Squamous Cell Carcinoma. Front. Cell. Infect. Microbiol..

[38] Bhattacharyya A., Chattopadhyay R., Mitra S., Crowe S.E. (2014). Oxidative stress: An essential factor in the pathogenesis of gastrointestinal mucosal diseases. Physiol. Rev..

[39] Farzaei M.H., Abdollahi M., Rahimi R. (2015). Role of dietary polyphenols in the management of peptic ulcer. World J. Gastroenterol..

[40] Rao C.V., Vijayakumar M. (2008). Effect of quercetin, flavonoids and alpha-tocopherol, an antioxidant vitamin, on experimental reflux oesophagitis in rats. Eur. J. Pharmacol..

[41] Nelkine L., Vrolijk M.F., Drent M., Bast A. (2020). Role of antioxidants in the treatment of gastroesophageal reflux disease-associated idiopathic pulmonary fibrosis. Curr. Opin. Pulm. Med..

[42] Gurlek I.K., Muderrisoglu A., Er Z.C., Arici A., Kupeli M. (2024). Evaluation of effects of curcumin on acute esophagitis in the corrosive esophagitis model in rats. Naunyn-Schmiedeberg’s Arch. Pharmacol..

[43] Oz H.S. (2017). Chronic Inflammatory Diseases and Green Tea Polyphenols. Nutrients.

[44] Mokra D., Joskova M., Mokry J. (2022). Therapeutic Effects of Green Tea Polyphenol (–)-Epigallocatechin-3-Gallate (EGCG) in Relation to Molecular Pathways Controlling Inflammation, Oxidative Stress, and Apoptosis. Int. J. Mol. Sci..

[45] Lee H.C., Jenner A.M., Low C.S., Lee Y.K. (2006). Effect of tea phenolics and their aromatic fecal bacterial metabolites on intestinal microbiota. Res. Microbiol..

[46] Zhang W., Qi S., Xue X., Al Naggar Y., Wu L., Wang K. (2021). Understanding the Gastrointestinal Protective Effects of Polyphenols using Foodomics-Based Approaches. Front. Immunol..

[47] Cardona F., Andrés-Lacueva C., Tulipani S., Tinahones F.J., Queipo-Ortuño M.I. (2013). Benefits of polyphenols on gut microbiota and implications in human health. J. Nutr. Biochem..

[48] Gupta S.S., Azmi L., Mohapatra P.K., Rao C.V. (2017). Flavonoids from whole Plant of Euphorbia hirta and their Evaluation against Experimentally induced Gastroesophageal Reflux Disease in Rats. Pharmacogn. Mag..

[49] Santin J.R., Lemos M., Klein-Júnior L.C., Machado I.D., Costa P., de Oliveira A.P., Tilia C., de Souza J.P., de Sousa J.P., Bastos J.K. (2011). Gastroprotective activity of essential oil of the Syzygium aromaticum and its major component eugenol in different animal models. Naunyn-Schmiedeberg’s Arch. Pharmacol..

[50] Moraes T.M., Rozza A.L., Kushima H., Pellizzon C.H., Rocha L.R., Hiruma-Lima C.A. (2013). Healing actions of essential oils from Citrus aurantium and d-limonene in the gastric mucosa: The roles of VEGF, PCNA, and CO_X−2_ in cell proliferation. J. Med. Food.

[51] Rozza A.L., Moraes Tde M., Kushima H., Tanimoto A., Marques M.O., Bauab T.M., Hiruma-Lima C.A., Pellizzon C.H. (2011). Gastroprotective mechanisms of Citrus lemon (Rutaceae) essential oil and its majority compounds limonene and β-pinene: Involvement of heat-shock protein-70, vasoactive intestinal peptide, glutathione, sulfhydryl compounds, nitric oxide and prostaglandin E_2_. Chem.-Biol. Interact..

[52] Caldas G.F., Oliveira A.R., Araújo A.V., Quixabeira D.C., Silva-Neto Jda C., Costa-Silva J.H., de Menezes I.R., Ferreira F., Leite A.C., da Costa J.G. (2014). Gastroprotective and ulcer healing effects of essential oil of Hyptis martiusii Benth. (Lamiaceae). PLoS ONE.

[53] Srivastava J.K., Shankar E., Gupta S. (2010). Chamomile: A herbal medicine of the past with bright future. Mol. Med. Rep..

[54] Habeeb F., Stables G., Bradbury F., Nong S., Cameron P., Plevin R., Ferro V.A. (2007). The inner gel component of Aloe vera suppresses bacterial-induced pro-inflammatory cytokines from human immune cells. Methods.

[55] Pastorino G., Cornara L., Soares S., Rodrigues F., Oliveira M. (2018). Liquorice (*Glycyrrhiza glabra*): A phytochemical and pharmacological review. Phytother. Res. PTR.

[56] Murray M.T. (2020). Glycyrrhiza Glabra (Licorice). Textbook of Natural Medicine.

[57] Ghayur M.N., Gilani A.H. (2006). Species differences in the prokinetic effects of ginger. Int. J. Food Sci. Nutr..

[58] Yeh A.M., Golianu B. (2014). Integrative Treatment of Reflux and Functional Dyspepsia in Children. Children.

[59] Murck H. (2020). Symptomatic Protective Action of Glycyrrhizin (Licorice) in COVID-19 Infection?. Front. Immunol..

[60] El-Saber Batiha G., Magdy Beshbishy A., El-Mleeh A., Abdel-Daim M.M., Prasad Devkota H. (2020). Traditional Uses, Bioactive Chemical Constituents, and Pharmacological and Toxicological Activities of *Glycyrrhiza glabra* L. (Fabaceae). Biomolecules.

[61] Hardy H., Harris J., Lyon E., Beal J., Foey A.D. (2013). Probiotics, prebiotics and immunomodulation of gut mucosal defences: Homeostasis and immunopathology. Nutrients.

[62] Keikha M., Karbalaei M. (2021). Probiotics as the live microscopic fighters against Helicobacter pylori gastric infections. BMC Gastroenterol..

[63] Chen Y.H., Tsai W.H., Wu H.Y., Chen C.Y., Yeh W.L., Chen Y.H., Hsu H.Y., Chen W.W., Chen Y.W., Chang W.W. (2019). Probiotic *Lactobacillus* spp. act Against Helicobacter pylori-induced Inflammation. J. Clin. Med..

[64] Chaudhry T.S., Senapati S.G., Gadam S., Mannam H., Voruganti H.V., Abbasi Z., Abhinav T., Challa A.B., Pallipamu N., Bheemisetty N. (2023). The Impact of Microbiota on the Gut-Brain Axis: Examining the Complex Interplay and Implications. J. Clin. Med..

[65] Davani-Davari D., Negahdaripour M., Karimzadeh I., Seifan M., Mohkam M., Masoumi S.J., Berenjian A., Ghasemi Y. (2019). Prebiotics: Definition, Types, Sources, Mechanisms, and Clinical Applications. Foods.

[66] Weh K.M., Howard C.L., Zhang Y., Tripp B.A., Clarke J.L., Howell A.B., Rubenstein J.H., Abrams J.A., Westerhoff M., Kresty L.A. (2024). Prebiotic proanthocyanidins inhibit bile reflux–induced esophageal adenocarcinoma through reshaping the gut microbiome and esophageal metabolome. JCI Insight.

[67] Zhang M., Hou Z.K., Huang Z.B., Chen X.L., Liu F.B. (2021). Dietary and Lifestyle Factors Related to Gastroesophageal Reflux Disease: A Systematic Review. Ther. Clin. Risk Manag..

[68] Özenoğlu A., Anul N., Özçelikçi B. (2023). The relationship of gastroesophageal reflux with nutritional habits and mental disorders. Hum. Nutr. Metab..

[69] Zalvan C.H., Hu S., Greenberg B., Geliebter J. (2017). A Comparison of Alkaline Water and Mediterranean Diet vs Proton Pump Inhibition for Treatment of Laryngopharyngeal Reflux. JAMA Otolaryngol. Head Neck Surg..

[70] Heidarzadeh-Esfahani N., Soleimani D., Hajiahmadi S., Moradi S., Heidarzadeh N., Nachvak S.M. (2021). Dietary Intake in Relation to the Risk of Reflux Disease: A Systematic Review. Prev. Nutr. Food Sci..

[71] Koufman J.A., Johnston N. (2012). Potential benefits of pH 8.8 alkaline drinking water as an adjunct in the treatment of reflux disease. Ann. Otol. Rhinol. Laryngol..

[72] Hungin A.P., Yadlapati R., Anastasiou F., Bredenoord A.J., El Serag H., Fracasso P., Mendive J.M., Savarino E.V., Sifrim D., Udrescu M. (2024). Management advice for patients with reflux-like symptoms: An evidence-based consensus. Eur. J. Gastroenterol. Hepatol..

[73] Gyawali C.P., Fass R. (2018). Management of Gastroesophageal Reflux Disease. Gastroenterology.

[74] Zdrhova L., Bitnar P., Balihar K., Kolar P., Madle K., Martinek M., Pandolfino J.E., Martinek J. (2023). Breathing Exercises in Gastroesophageal Reflux Disease: A Systematic Review. Dysphagia.

[75] Bonaterra G.A., Bronischewski K., Hunold P., Schwarzbach H., Heinrich E.U., Fink C., Aziz-Kalbhenn H., Müller J., Kinscherf R. (2020). Anti-inflammatory and Anti-oxidative Effects of Phytohustil(^®^) and Root Extract of Althaea officinalis L. on Macrophages in vitro. Front. Pharmacol..

[76] Ahuja A., Ahuja N.K. (2019). Popular Remedies for Esophageal Symptoms: A Critical Appraisal. Curr. Gastroenterol. Rep..

[77] Bang C.S., Yang Y.J., Baik G.H. (2019). Melatonin for the treatment of gastroesophageal reflux disease; protocol for a systematic review and meta-analysis. Medicine.

[78] Malekpour H., Noori A., Abdi S., Abbasinazari M., Mahboubi A., Ghamsari M.A. (2023). Is the Addition of Sublingual Melatonin to Omeprazole Superior to Omeprazole Alone in the Management of Gastroesophageal Reflux Disease Symptoms: A Clinical Trial. Turk. J. Gastroenterol. Off. J. Turk. Soc. Gastroenterol..

[79] Jabri M.A., Tounsi H., Rtibi K., Marzouki L., Sakly M., Sebai H. (2016). Ameliorative and antioxidant effects of myrtle berry seed (*Myrtus communis*) extract during reflux-induced esophagitis in rats. Pharm. Biol..

[80] Naeimi M., Kianifar H., Memariani Z., Kamalinejad M., Bijani A., Saghebi R., Gorji N. (2019). Comparison of the efficacy of ranitidine and quince syrup on gastroesophageal reflux disease in children. Complement. Ther. Med..

[81] Nam Y., Lee J.M., Wang Y., Ha H.S., Sohn U.D. (2016). The effect of Flos Lonicerae Japonicae extract on gastro-intestinal motility function. J. Ethnopharmacol..

[82] Kim Y.S., Kim J.-W., Ha N.-Y., Kim J., Ryu H.S. (2020). Herbal Therapies in Functional Gastrointestinal Disorders: A Narrative Review and Clinical Implication. Front. Psychiatry.

[83] Kwiatek M.A., Roman S., Fareeduddin A., Pandolfino J.E., Kahrilas P.J. (2011). An alginate-antacid formulation (Gaviscon Double Action Liquid) can eliminate or displace the postprandial ‘acid pocket’ in symptomatic GERD patients. Aliment. Pharmacol. Ther..

[84] Leiman D.A., Riff B.P., Morgan S., Metz D.C., Falk G.W., French B., Umscheid C.A., Lewis J.D. (2017). Alginate therapy is effective treatment for GERD symptoms: A systematic review and meta-analysis. Dis. Esophagus Off. J. Int. Soc. Dis. Esophagus.

[85] Schulz R.M., Ahuja N.K., Slavin J.L. (2022). Effectiveness of Nutritional Ingredients on Upper Gastrointestinal Conditions and Symptoms: A Narrative Review. Nutrients.

[86] de Souza M.C., Vieira A.J., Beserra F.P., Pellizzon C.H., Nóbrega R.H., Rozza A.L. (2019). Gastroprotective effect of limonene in rats: Influence on oxidative stress, inflammation and gene expression. Phytomedicine.

[87] Cho J.H., Yoon H., Shin C.M., Park Y.S., Kim N., Lee D.H. (2020). Efficacy of DA-5204 (Stillen 2X) for patients with gastroesophageal reflux disease: A randomized, double-blind, placebo-controlled pilot study. Medicine.

[88] Ding Y., Brand E., Wang W., Zhao Z. (2022). Licorice: Resources, applications in ancient and modern times. J. Ethnopharmacol..

[89] Smedegaard S.B., Svart M.V. (2019). Licorice induced pseudohyperaldosteronism, severe hypertension, and long QT. Endocrinol. Diabetes Metab. Case Rep..

[90] Raveendra K.R., Jayachandra Srinivasa V., Sushma K.R., Allan J.J., Goudar K.S., Shivaprasad H.N., Venkateshwarlu K., Geetharani P., Sushma G., Agarwal A. (2012). An Extract of Glycyrrhiza glabra (GutGard) Alleviates Symptoms of Functional Dyspepsia: A Randomized, Double-Blind, Placebo-Controlled Study. Evid.-Based Complement. Altern. Med..

[91] Raj J.P., Saxena U., Belhekar M.N., Mamde A., Darak H., Pawar S. (2025). Efficacy and Safety of GutGard^®^ in Managing Gastroesophageal Reflux-Related Symptoms: A Phase III, Single-Centre, Double-Blind, Randomized Placebo-Controlled Trial. Complement. Med. Res..

[92] Wahab S., Annadurai S., Abullais S.S., Das G., Ahmad W., Ahmad M.F., Kandasamy G., Vasudevan R., Ali M.S., Amir M. (2021). Glycyrrhiza glabra (Licorice): A Comprehensive Review on Its Phytochemistry, Biological Activities, Clinical Evidence and Toxicology. Plants.

[93] Setright R. (2017). Prevention of symptoms of gastric irritation (GERD) using two herbal formulas: An observational study. J. Aust. Tradit.-Med. Soc..

[94] Li Z., Tao L., Zhang S.S., Sun X.H., Chen S.N., Wu J. (2021). Modified Xiaochaihu Decoction for gastroesophageal reflux disease: A randomized double-simulation controlled trial. World J. Gastroenterol..

[95] Panahi Y., Khedmat H., Valizadegan G., Mohtashami R., Sahebkar A. (2015). Efficacy and safety of Aloe vera syrup for the treatment of gastroesophageal reflux disease: A pilot randomized positive-controlled trial. J. Tradit. Chin. Med. = Chung I Tsa Chih Ying Wen Pan.

[96] Basu P., Hempole H., Krishnaswamy N., Shah N., Aloysius M. (2014). The effect of melatonin in functional heartburn: A randomized, placebo-controlled clinical trial. Open J. Gastroenterol..

[97] Zohalinezhad M.E., Hosseini-Asl M.K., Akrami R., Nimrouzi M., Salehi A., Zarshenas M.M. (2015). *Myrtus communis* L. Freeze-Dried Aqueous Extract Versus Omeprazol in Gastrointestinal Reflux Disease: A Double-Blind Randomized Controlled Clinical Trial. J. Evid.-Based Complement. Altern. Med..

[98] Paknejad M.S., Eftekhari K., Rahimi R., Vigeh M., Naghizadeh A., Karimi M. (2021). Myrtle (*Myrtus communis* L.) fruit syrup for gastroesophageal reflux disease in children: A double-blind randomized clinical trial. Phytother. Res. PTR.

[99] Tseng W.H., Tseng P.H., Wu J.F., Hsu Y.C., Lee T.Y., Ni Y.H., Wang H.P., Hsiao T.Y., Hsu W.C. (2018). Double-blind, placebo-controlled study with alginate suspension for laryngopharyngeal reflux disease. Laryngoscope.

[100] Reimer C., Lødrup A.B., Smith G., Wilkinson J., Bytzer P. (2016). Randomised clinical trial: Alginate (Gaviscon Advance) vs. placebo as add-on therapy in reflux patients with inadequate response to a once daily proton pump inhibitor. Aliment. Pharmacol. Ther..

[101] Kim J.H., Lee Y.C., Kim E.H., Park J.C., Shin S.K., Lee S.K., Jung D.H., Park J.J., Youn Y.H., Park H. (2019). The Clinical Efficacy of a Pure Alginate Formulation (Lamina G) for Controlling Symptoms in Individuals with Reflux Symptoms: A Randomized Clinical Study. Gut Liver.

[102] Alecci U., Bonina F., Bonina A., Rizza L., Inferrera S., Mannucci C., Calapai G. (2016). Efficacy and Safety of a Natural Remedy for the Treatment of Gastroesophageal Reflux: A Double-Blinded Randomized-Controlled Study. Evid.-Based Complement. Altern. Med. Ecam.

[103] Liu W., Xie Y., Li Y., Zheng L., Xiao Q., Zhou X., Li Q., Yang N., Zuo K., Xu T. (2022). Protocol of a randomized, double-blind, placebo-controlled study of the effect of probiotics on the gut microbiome of patients with gastro-oesophageal reflux disease treated with rabeprazole. BMC Gastroenterol..

[104] Wu Y., Guo Y., Huang T., Huang D., Liu L., Shen C., Jiang C., Wang Z., Chen H., Liang P. (2023). Licorice flavonoid alleviates gastric ulcers by producing changes in gut microbiota and promoting mucus cell regeneration. Biomed. Pharmacother..

[105] Ling W., Huang Y., Xu J.H., Li Y., Huang Y.M., Ling H.B., Sui Y., Zhao H.L. (2015). Consistent Efficacy of Wendan Decoction for the Treatment of Digestive Reflux Disorders. Am. J. Chin. Med..

[106] Shih Y.S., Tsai C.H., Li T.C., Yu C.J., Chou J.W., Feng C.L., Wang K.T., Lai H.C., Hsieh C.L. (2019). Effect of wu chu yu tang on gastroesophageal reflux disease: Randomized, double-blind, placebo-controlled trial. Phytomedicine.

[107] Li S., Huang M., Wu G., Huang W., Huang Z., Yang X., Ou J., Wei Q., Liu C., Yu S. (2020). Efficacy of Chinese Herbal Formula Sini Zuojin Decoction in Treating Gastroesophageal Reflux Disease: Clinical Evidence and Potential Mechanisms. Front. Pharmacol..

[108] Xu J.H., Huang Y.M., Ling W., Li Y., Wang M., Chen X.Y., Sui Y., Zhao H.L. (2015). Wen Dan Decoction for hemorrhagic stroke and ischemic stroke. Complement. Ther. Med..

[109] Pan S., Li J., Zhang X., Li Y., Yan J., Zhang W., Hu S. (2017). [Clinical trial of gastroesophageal reflux disease with the disharmony between liver and stomach syndrome treated with acupuncture regulating *qi* based on the compatibility of the five meridians]. Zhongguo Zhen Jiu = Chin. Acupunct. Moxibustion.

[110] Hsu C.S., Wang C.C., Wang P.C., Lin H.H., Tseng T.C., Chen C.H., Su W.C., Liu C.J., Chen C.L., Lai M.Y. (2010). Increased incidence of gastroesophageal reflux disease in patients with chronic hepatitis B virus infection. Hepatol. Int..

[111] Chen C., Gong X., Yang X., Shang X., Du Q., Liao Q., Xie R., Chen Y., Xu J. (2019). The roles of estrogen and estrogen receptors in gastrointestinal disease. Oncol. Lett..

[112] Baser K.H., Demirci B., Iscan G., Hashimoto T., Demirci F., Noma Y., Asakawa Y. (2006). The essential oil constituents and antimicrobial activity of Anthemis aciphylla BOISS. var. discoidea BOISS. Chem. Pharm. Bull..

[113] McKay D.L., Blumberg J.B. (2006). A review of the bioactivity and potential health benefits of chamomile tea (*Matricaria recutita* L.). Phytother. Res. PTR.

[114] Crotteau C.A., Wright S.T., Eglash A. (2006). Clinical inquiries. What is the best treatment for infants with colic?. J. Fam. Pract..

[115] Aregawi L.G., Shokrolahi M., Gebremeskel T.G., Zoltan C. (2023). The Effect of Ginger Supplementation on the Improvement of Dyspeptic Symptoms in Patients With Functional Dyspepsia. Cureus.

[116] Samota M.K., Rawat M., Kaur M., Garg D. (2024). Gingerol: Extraction methods, health implications, bioavailability and signaling pathways. Sustain. Food Technol..

[117] Nikkhah Bodagh M., Maleki I., Hekmatdoost A. (2019). Ginger in gastrointestinal disorders: A systematic review of clinical trials. Food Sci. Nutr..

[118] Anh N.H., Kim S.J., Long N.P., Min J.E., Yoon Y.C., Lee E.G., Kim M., Kim T.J., Yang Y.Y., Son E.Y. (2020). Ginger on Human Health: A Comprehensive Systematic Review of 109 Randomized Controlled Trials. Nutrients.

[119] Hirata A., Funato H., Nakai M., Iizuka M., Abe N., Yagi Y., Shiraishi H., Jobu K., Yokota J., Hirose K. (2016). Ginger Orally Disintegrating Tablets to Improve Swallowing in Older People. Biol. Pharm. Bull..

[120] Palatty P.L., Haniadka R., Valder B., Arora R., Baliga M.S. (2013). Ginger in the prevention of nausea and vomiting: A review. Crit. Rev. Food Sci. Nutr..

[121] Mohd Yusof Y.A. (2016). Gingerol and Its Role in Chronic Diseases. Adv. Exp. Med. Biol..

[122] Farhat C., Younes H., Alyamani O.A., Mrad M., Hourani N., Khalifeh H., El-Makhour Y., Dbaibo G., Hage-Sleiman R. (2022). Chemical characterization and in vitro biological evaluation of aqueous extract of Althaea officinalis L. flower grown in Lebanon. J. Herb. Med..

[123] Deters A., Zippel J., Hellenbrand N., Pappai D., Possemeyer C., Hensel A. (2010). Aqueous extracts and polysaccharides from Marshmallow roots (*Althea officinalis* L.): Cellular internalisation and stimulation of cell physiology of human epithelial cells in vitro. J. Ethnopharmacol..

[124] Zaghlool S.S., Abo-Seif A.A., Rabeh M.A., Abdelmohsen U.R., Messiha B.A.S. (2019). Gastro-Protective and Anti-Oxidant Potential of Althaea officinalis and Solanum nigrum on Pyloric Ligation/Indomethacin-Induced Ulceration in Rats. Antioxidants.

[125] Hage-Sleiman R., Mroueh M., Daher C.F. (2011). Pharmacological evaluation of aqueous extract of Althaea officinalis flower grown in Lebanon. Pharm. Biol..

[126] Pallardy S.G. (2007). Physiology of Woody Plants.

[127] Upton P., Axentiev P., Swisher D. (2011). American Herbal Pharmacopoeia and Therapeutic Compendium: Slippery Elm Inner Bark (Ulmus rubra Muhl.)—Standards of Analysis, Quality Control, and Therapeutics Monograph.

[128] Ried K., Travica N., Dorairaj R., Sali A. (2020). Herbal formula improves upper and lower gastrointestinal symptoms and gut health in Australian adults with digestive disorders. Nutr. Res..

[129] Kumar R., Singh A.K., Gupta A., Bishayee A., Pandey A.K. (2019). Therapeutic potential of Aloe vera—A miracle gift of nature. Phytomedicine.

[130] Catalano A., Ceramella J., Iacopetta D., Marra M., Conforti F., Lupi F.R., Gabriele D., Borges F., Sinicropi M.S. (2024). Aloe vera―An Extensive Review Focused on Recent Studies. Foods.

[131] Yu M., Kong X.Y., Chen T.T., Zou Z.M. (2024). In vivo metabolism combined network pharmacology to identify anti-constipation constituents in Aloe barbadensis Mill. J. Ethnopharmacol..

[132] Keshavarzi Z., Rezapour T.M., Vatanchian M., Zare Hesari M., Nabizade Haghighi H., Izanlu M., Sabaghian M., Shahveisi K. (2014). The effects of aqueous extract of Aloe vera leaves on the gastric acid secretion and brain and intestinal water content following acetic acid- induced gastric ulcer in male rats. Avicenna J. Phytomed..

[133] Mahboubi M. (2021). Aloe Vera (*Aloe barbadensis*) Gel for the Management of Gastroesophageal Reflux Disease (GERD). Nat. Prod. J..

[134] Cheung T., Hailemeskel B., Fullas F. (2024). A survey on the treatment of GERD with aloe vera juice, slippery elm, ginger tea, and chamomile tea. GSC Biol. Pharm. Sci..

[135] Langmead L., Feakins R.M., Goldthorpe S., Holt H., Tsironi E., De Silva A., Jewell D.P., Rampton D.S. (2004). Randomized, double-blind, placebo-controlled trial of oral aloe vera gel for active ulcerative colitis. Aliment. Pharmacol. Ther..

[136] Zhang L.-J., Huang X.-J., Shi X.-D., Chen H.-H., Cui S.W., Nie S.-P. (2019). Protective effect of three glucomannans from different plants against DSS induced colitis in female BALB/c mice. Food Funct..

[137] Ismaeil H., Abdo W., Amer S., Tahoun A., Massoud D., Zanaty E., Bin-Jumah M., Mahmoud A.M. (2024). Correction: Ismaeil et al. Ameliorative Effect of Heat-Killed Lactobacillus plantarum L.137 and/or Aloe vera against Colitis in Mice. *Processes*
**2020**, *8*, 225. Processes.

[138] Sangil-Monroy M., Serra-Majem L.s., Monroy J.M.M., Andrellucchi A.O., S¨¢nchez-Villegas A., Doreste J., Knipschild P. (2014). Effects of Intake of Milk Enriched with *Aloe vera* on Patients with Gastrointestinal Reflux Disease. Food Nutr. Sci..

[139] Majka J., Wierdak M., Brzozowska I., Magierowski M., Szlachcic A., Wojcik D., Kwiecien S., Magierowska K., Zagajewski J., Brzozowski T. (2018). Melatonin in Prevention of the Sequence from Reflux Esophagitis to Barrett’s Esophagus and Esophageal Adenocarcinoma: Experimental and Clinical Perspectives. Int. J. Mol. Sci..

[140] Minich D.M., Henning M., Darley C., Fahoum M., Schuler C.B., Frame J. (2022). Is Melatonin the “Next Vitamin D”?: A Review of Emerging Science, Clinical Uses, Safety, and Dietary Supplements. Nutrients.

[141] Celinski K., Konturek P.C., Konturek S.J., Slomka M., Cichoz-Lach H., Brzozowski T., Bielanski W. (2011). Effects of melatonin and tryptophan on healing of gastric and duodenal ulcers with Helicobacter pylori infection in humans. J. Physiol. Pharmacol. Off. J. Pol. Physiol. Soc..

[142] Meng X., Li Y., Li S., Zhou Y., Gan R.Y., Xu D.P., Li H.B. (2017). Dietary Sources and Bioactivities of Melatonin. Nutrients.

[143] Yahyazadeh R., Baradaran Rahimi V., Yahyazadeh A., Mohajeri S.A., Askari V.R. (2021). Promising effects of gingerol against toxins: A review article. BioFactors.

[144] Dabbaghi M.M., Fadaei M.S., Soleimani Roudi H., Baradaran Rahimi V., Askari V.R. (2023). A review of the biological effects of Myrtus communis. Physiol. Rep..

[145] Shakeri A., Hashempur M.H., Mojibian M., Aliasl F., Bioos S., Nejatbakhsh F. (2018). A comparative study of ranitidine and quince (*Cydonia oblonga* mill) sauce on gastroesophageal reflux disease (GERD) in pregnancy: A randomised, open-label, active-controlled clinical trial. J. Obstet. Gynaecol. J. Inst. Obstet. Gynaecol..

[146] Parvan M., Sajjadi S.E., Minaiyan M. (2017). Protective Effect of Two Extracts of Cydonia oblonga Miller (Quince) Fruits on Gastric Ulcer Induced by Indomethacin in Rats. Int. J. Prev. Med..

[147] Ku S.K., Seo B.I., Park J.H., Park G.Y., Seo Y.B., Kim J.S., Lee H.S., Roh S.S. (2009). Effect of Lonicerae Flos extracts on reflux esophagitis with antioxidant activity. World J. Gastroenterol..

[148] Choi Y., Kim N., Noh G.T., Lee J.Y., Lee D.H. (2020). The Efficacy and Safety of GCWB104 (Flos Lonicera Extract) in Functional Dyspepsia: A Single-Center, Randomized, Double-Blind, Placebo-Controlled Study. Gut Liver.

[149] Kang J.W., Yun N., Han H.J., Kim J.Y., Kim J.Y., Lee S.M. (2014). Protective Effect of Flos Lonicerae against Experimental Gastric Ulcers in Rats: Mechanisms of Antioxidant and Anti-Inflammatory Action. Evid.-Based Complement. Altern. Med. Ecam.

[150] Pilichiewicz A.N., Horowitz M., Russo A., Maddox A.F., Jones K.L., Schemann M., Holtmann G., Feinle-Bisset C. (2007). Effects of Iberogast on proximal gastric volume, antropyloroduodenal motility and gastric emptying in healthy men. Am. J. Gastroenterol..

[151] Oude Nijhuis R.A.B., Kuipers T., Oors J.M., Herregods T.V.K., Kessing B.F., Schuitenmaker J.M., Smout A., Bredenoord A.J. (2024). The Effect of STW5 (Iberogast) on Reflux Symptoms in Patients With Concurrent Dyspeptic Symptoms: A Double-blind Randomized Placebo-controlled Crossover Trial. J. Neurogastroenterol. Motil..

[152] Michael R., Bettina V., Eckehard L. (2022). Functional gastrointestinal disorders in children: Effectivity, safety, and tolerability of the herbal preparation STW-5 (Iberogast^®^) in general practice. Complement. Ther. Med..

[153] Khayyal M.T., Seif-El-Nasr M., El-Ghazaly M.A., Okpanyi S.N., Kelber O., Weiser D. (2006). Mechanisms involved in the gastro-protective effect of STW 5 (Iberogast) and its components against ulcers and rebound acidity. Phytomedicine.

[154] Wadie W., Abdel-Aziz H., Zaki H.F., Kelber O., Weiser D., Khayyal M.T. (2012). STW 5 is effective in dextran sulfate sodium-induced colitis in rats. Int. J. Color. Dis..

[155] Dossett M.L., Cohen E.M., Cohen J. (2017). Integrative Medicine for Gastrointestinal Disease. Prim. Care.

[156] Quartarone G. (2013). Gastroesophageal reflux in pregnancy: A systematic review on the benefit of raft forming agents. Minerva Ginecol..

[157] Senthil Kumar K.J., Gokila Vani M., Dakpa G., Wang S.Y. (2025). Dietary limonene promotes gastrointestinal barrier function via upregulating tight/adherens junction proteins through cannabinoid receptor type-1 antagonistic mechanism and alters cellular metabolism in intestinal epithelial cells. BioFactors.

[158] Kim J.M., Choi S.M., Kim D.H., Oh T.Y., Ahn B.O., Kwon J.W., Kim W.B. (2005). Combined use of omeprazole and a novel antioxidative cytoprotectant for the treatment of peptic ulcer. Facilitation of ulcer healing in experimental animals. Arzneimittelforschung.

[159] Yarnell E., Abascal K. (2010). Herbs for Gastroesophageal Reflux Disease. Altern. Complement. Ther..

[160] Oh T.Y., Lee J.S., Ahn B.O., Cho H., Kim W.B., Kim Y.B., Surh Y.J., Cho S.W., Lee K.M., Hahm K.B. (2001). Oxidative stress is more important than acid in the pathogenesis of reflux oesophagitis in rats. Gut.

[161] Patrick L. (2011). Gastroesophageal reflux disease (GERD): A review of conventional and alternative treatments. Altern. Med. Rev..

[162] Seol S.Y., Kim M.H., Ryu J.S., Choi M.G., Shin D.W., Ahn B.O. (2004). DA-9601 for erosive gastritis: Results of a double-blind placebo-controlled phase III clinical trial. World J. Gastroenterol..

[163] Jeong D., Yi Y.S., Sung G.H., Yang W.S., Park J.G., Yoon K., Yoon D.H., Song C., Lee Y., Rhee M.H. (2014). Anti-inflammatory activities and mechanisms of Artemisia asiatica ethanol extract. J. Ethnopharmacol..

[164] Bidian C., Mitrea D.R., Vasile O.G., Filip A., Cătoi A.F., Moldovan R., Decea N., Albu A. (2020). Quercetin and curcumin effects in experimental pleural inflammation. Med. Pharm. Rep..

[165] Min Y.S., Lee S.E., Hong S.T., Kim H.S., Choi B.C., Sim S.S., Whang W.K., Sohn U.D. (2009). The Inhibitory Effect of Quercetin-3-O-beta-D-Glucuronopyranoside on Gastritis and Reflux Esophagitis in Rats. Korean J. Physiol. Pharmacol. Off. J. Korean Physiol. Soc. Korean Soc. Pharmacol..

[166] Mahattanadul S., Radenahmad N., Phadoongsombut N., Chuchom T., Panichayupakaranant P., Yano S., Reanmongkol W. (2006). Effects of curcumin on reflux esophagitis in rats. J. Nat. Med..

[167] Lee J.A., Shin M.R., Kim M.J., Lee J.H., Park H.J., Roh S.S. (2021). Protective Effects of Inflammation of Curcumae Longae Rhizoma 30% EtOH Extract on Acute Reflux Esophagitis Rats. BioMed Res. Int..

[168] Bozomitu L., Miron I., Adam Raileanu A., Lupu A., Paduraru G., Marcu F.M., Buga A.M.L., Rusu D.C., Dragan F., Lupu V.V. (2022). The Gut Microbiome and Its Implication in the Mucosal Digestive Disorders. Biomedicines.

[169] Yoshida N. (2007). Inflammation and oxidative stress in gastroesophageal reflux disease. J. Clin. Biochem. Nutr..

[170] Amir I., Konikoff F.M., Oppenheim M., Gophna U., Half E.E. (2014). Gastric microbiota is altered in oesophagitis and Barrett’s oesophagus and further modified by proton pump inhibitors. Environ. Microbiol..

[171] Zhou J., Shrestha P., Qiu Z., Harman D.G., Teoh W.C., Al-Sohaily S., Liem H., Turner I., Ho V. (2020). Distinct Microbiota Dysbiosis in Patients with Non-Erosive Reflux Disease and Esophageal Adenocarcinoma. J. Clin. Med..

[172] Komolafe K., Komolafe T.R., Fatoki T.H., Akinmoladun A.C., Brai B.I.C., Olaleye M.T., Akindahunsi A.A. (2021). Coronavirus Disease 2019 and Herbal Therapy: Pertinent Issues Relating to Toxicity and Standardization of Phytopharmaceuticals. Rev. Bras. Farmacogn. Orgao Of. Soc. Bras. Farmacogn..

[173] Valussi M. (2012). Functional foods with digestion-enhancing properties. Int. J. Food Sci. Nutr..

